# Genomic Characterization of a Novel Yezo Virus Revealed in *Ixodes pavlovskyi* Tick Virome in Western Siberia

**DOI:** 10.3390/v17101362

**Published:** 2025-10-11

**Authors:** Maxim Apanasevich, Nikita Dubovitskiy, Anastasiya Derko, Anna Khozyainova, Alexander Tarasov, Alina Kokhanenko, Gleb Artemov, Evgeny Denisov, Alexander Shestopalov, Kirill Sharshov

**Affiliations:** 1 Research Institute of Virology, Federal Research Center of Fundamental and Translational Medicine, 630060 Novosibirsk, Russiasharshov@yandex.ru (K.S.); 2 Laboratory of Evolutionary Cytogenetics, Tomsk State University, 634050 Tomsk, Russia; 3 Institute of Medicine and Medical Technologies, Novosibirsk State University, 630090 Novosibirsk, Russia; 4 Cancer Research Institute, Tomsk National Research Medical Center, Russian Academy of Sciences, 634009 Tomsk, Russia; 5 Research Institute of Molecular and Cellular Medicine, Peoples’ Friendship University of Russia, 117198 Moscow, Russia

**Keywords:** virome, metagenomic sequencing, tick-borne viruses, *Ixodes pavlovskyi*, *Orthonaitovirus yezoense*

## Abstract

Ixodid ticks are blood-sucking ectoparasites of vertebrates. They constitute an integral part of natural foci and are responsible for the worldwide transmission of infections to humans, which can result in severe symptoms. For instance, the Tomsk region, where three abundant tick species (*Dermacentor reticulatus*, *Ixodes pavlovskyi*, *I. persulcatus*) occur, is an endemic area for tick-borne encephalitis virus (TBEV). An increasing number of novel infectious agents carried by ticks have been identified using metagenomic sequencing. A notable example is the Yezo virus (*Orthonairovirus yezoense*, YEZV), which was discovered in patients with fever after tick bites in Japan and China between 2014 and 2025. For the first time, we have performed metagenomic sequencing of the virome of ticks collected in the Tomsk region. In a sample obtained from a pool of *I. pavlovskyi* ticks, all three segments of the YEZV genome were detected. The phylogenetic analysis showed that the newly identified isolate formed a sister group to previously described virus isolates, indicating the presence of a new genetic variant. This study presents the first report of YEZV detection in *I. pavlovskyi* ticks in the Tomsk region, thereby expanding the geographical range and number of vector species for YEZV and highlighting the importance of monitoring viral agents circulating among ticks in Western Siberia.

## 1. Introduction

Ticks (order Ixodida) are divided into three main families: Ixodidae (722 species), Argasidae (208 species), and Nutalliellidae (1 species) [1,2]. Hard ticks, belonging to the family Ixodidae, are highly specialized obligate blood-sucking ectoparasites of terrestrial vertebrates, primarily birds and mammals and, to a lesser extent, reptiles [3,4]. Ticks are important vectors of various pathogenic microorganisms, including bacteria, viruses, and protozoa, and constitute one of the three main components of natural and synanthropic foci of transmissible infections [5]. Three abundant ixodid tick species have endemic significance in the Tomsk region: *D. reticulatus*, *I. pavlovskyi*, and *I. persulcatus* [6]. All of them, to varying degrees, participate in the spread of both well-known and regularly detected pathogens (*Anaplasma phagocytophilum*, *Babesia* spp., *Bartonella* spp., *Borrelia burgdorferi* s.l., *B. miyamotoi*, *Ehrlichia chaffeensis*, *E. muris*, *Francisella tularensis*, *Rickettsia* spp., TBEV, and West Nile virus), as well as newly discovered ones (viruses of the genera *Phlebovirus*, *Uukivirus*, and YEZV) [7,8,9,10]. In Russia, an analysis of 5318 *I. persulcatus* and *I. ricinus* ticks collected in 23 regions revealed five YEZV isolates in *I. persulcatus* ticks from three regions: Khabarovsk, Primorsky and Transbaikal territories [10]. However, within foci of tick-borne infections located in anthropogenically transformed areas, *I. pavlovskyi* is more significant, currently dominating the urban biotopes of Tomsk over both *I. persulcatus* and *D. reticulatus* [11]. Its ornithophilicity has been observed and documented at almost all life cycle stages (larva, nymph, imago) [12].

Given the wide range of pathogens carried by ixodid ticks, the identification of novel infectious agents capable of causing disease in humans and animals is of particular interest. A notable example is YEZV (*Orthonairovirus yezoense*), a recently identified member of the genus *Orthonairovirus* (*Bunyavirales*, *Nairoviridae*), first detected on Hokkaido Island, Japan, in 2019 [13]. The presence of YEZV has been confirmed in ixodid ticks (*D. silvarum*, *Haemaphysalis megaspinosa*, *H. japonica*, *I. ovatus* and *I. persulcatus*), in wild animals (*Cervus nippon yesoensis*, *Emberiza spodocephala* and *Procyon lotor*), and in the blood of 10 patients from Japan (since 2014) and 19 from China [10,13,14,15,16,17,18,19]. To date, YEZV has not been reported in *I. pavlovskyi*.

The clinical manifestations of YEZV infection in patients from Japan were more pronounced than in those from China and included temperature up to 39 °C, loss of appetite, leukopenia, lymphocytopenia, thrombocytopenia, and elevated levels of enzymes (alanine aminotransferase, aspartate transaminase, creatine kinase and lactate dehydrogenase) and ferritin, suggesting possible liver damage. Cases of blood coagulation disorders were also identified. In contrast, the disease in the Chinese patients presented in a milder form. In addition to elevated body temperature, changes in blood cell counts, and increased liver enzyme activity, clinical features such as headache, dizziness, joint pain, visual disturbances, shortness of breath, and fatigue were observed [13,14,16,18]. According to Matsuno [20], the disease manifested as severe in eight of 18 patients. Gastrointestinal disorders were documented in 9 patients, a phenomenon not previously reported in the literature. Neurological symptoms were also observed in 5 patients, although their exact nature was not specified. The clinical course of YEZV infection appears to vary depending on factors such as patient age and medical history, co-infection, and concomitant medication use. Importantly, all patients infected with YEZV achieved full recovery within two to three weeks [13,14,16,18,20].

According to the most recent official classification, the genus *Orthonairovirus* (*Nairoviridae*) includes 51 species, for which argasid or ixodid ticks serve as vectors [21]. The genome of *Orthonairovirus* is characterized by three linear single-stranded negative-sense RNA (ssRNA (–)) segments, ranging in total size from 17.2 to 20.1 kb (S segment: 1.4–3.8 kb; M segment: 3.9–5.9 kb; L segment: 11.7–12.6 kb) [22]. Most members of the genus *Orthonairovirus* are capable of replicating in both arthropods (argasid and ixodid ticks) and vertebrate hosts [23]. The recent discovery of previously unknown viral agents in ixodid tick populations indicates that the spectrum of pathogens carried by these ectoparasites is far from fully understood [7,8,9,10]. This underscores the need for modern research methods that improve detection efficiency and enable rapid monitoring of new or re-emerging pathogens. A representative example of such a method is metagenomic next-generation sequencing [24], the use of which markedly increased over the past 15 years [25]. The objective of the present study was to perform the genomic characterization of a newly identified orthonairoviruses, designated YEZV, which was detected by metagenomic sequencing in female *I. pavlovskyi* ticks collected in Tomsk.

## 2. Materials and Methods

### 2.1. Sampling

The collection of female *I. pavlovskyi* was carried out in June 2024 in Tomsk (Tomsk region, Russia). Ixodid ticks were collected from vegetation using the standard flagging method. Each tick was placed individually into a 1.5 mL tube and transported to the laboratory alive, where they were stored at 4 °C until species identification. Using morphological keys [26], the species, sex, and developmental stage of each tick were determined under a binocular microscope. After identification, each tick was washed sequentially in 70% ethanol and sterile water, placed individually into a 0.5 mL tube, frozen in liquid nitrogen, and stored at −80 °C.

### 2.2. Enrichment of Virus-like Particles, Nucleic Acid Extraction, Library Preparation and Metagenomic Sequencing

The enrichment of virus-like particles was performed according to a modified NetoVir protocol [27]. Prior to homogenization, individual ixodid tick samples were combined into pools, resulting in two pools of eight *I. pavlovskyi* individuals each. A negative control consisting only of Hanks’ solution with phenol red (BioloT, Saint-Petersburg, Russia) was included for each batch of tick pools. Homogenization of the pools was carried out using 2.8 mm ceramic beads (Allsheng, Hangzhou, China) and Hank’s solution with phenol red (BioloT, Saint-Petersburg, Russia) on a Bioprep-24 instrument (Allsheng, Hangzhou, China) at 7000 rpm at room temperature for 90 s. This procedure was repeated 4 times, with tubes placed at −20 °C for 1 min between repeats. The pools were then centrifuged at 17,000× *g* at 3 °C for 3 min, and the supernatant was passed through a 0.8 µm pore-size centrifugal microfilter (Sartorius, Göttingen, Germany). The filtrate was treated with micrococcal nuclease (Thermo Fisher Scientific, Ipswich, MA, USA) and benzonase (diaGene, Moscow, Russia). Nucleic acids were extracted using a column-based RNA extraction kit (Biolabmix, Novosibirsk, Russia) according to the manufacturer’s protocol. Whole-transcriptome amplification was performed using the WTA2 kit (Sigma-Aldrich, St. Louis, MO, USA) according to the NetoVir protocol. The PCR product was purified using a DNA/RNA extraction kit (Biolabmix, Novosibirsk, Russia). PCR products from the two pools were then combined, and library preparation was initiated. Libraries for massively parallel sequencing were prepared using the SyntEra-DNA kit (Syntol, Moscow, Russia) according to the manufacturer’s protocol. Following library preparation, reconditioning PCR was performed for the combined sample using the SyntEra-DNA kit with standard Illumina P5 (5′-AATGATACGGCGACCGAGATCT-3′) and P7 (5′-CAAGCAGAAGGGCATACGAGAT-3′) primers and the Encyclo Plus PCR kit (Evrogen, Moscow, Russia). Two-sided size selection was conducted using AMPure XP magnetic beads (Beckman Coulter, Brea, CA, USA). The PCR reaction mixture consisted of 16 µL dH2O, 2.5 µL 10× Encyclo buffer, 0.5 µL 50× dNTP mix, 2.5 µL P7 and P5 primer mix, 0.5 µL 50× Encyclo polymerase mix, and 3 µL DNA. PCR conditions were as follows: initial denaturation at 95 °C for 2 min, 6 cycles of 95 °C for 15 s, 60 °C for 30 s, 72 °C for 30 s, and final elongation at 72 °C for 1 min. After reconditioning PCR and subsequent purification and size selection, fragment length analysis was performed on a TapeStation 4150 instrument (Agilent Technologies, Santa Clara, CA, USA). Prepared libraries were sequenced on a Genolab M platform (GeneMind Biosciences, Shenzhen, China) with 150-nucleotide paired-end reads, yielding an average of approximately 10 million reads per sample. Sequencing was conducted at the Core Facility “Medical genomics” of Tomsk National Research Medical Center of the Russian Academy of Sciences (Tomsk, Russia).

### 2.3. Virome Data Analysis

Raw reads were processed using the ViPER pipeline v2.3 [28]. Untreated raw reads were trimmed (ILLUMINACLIP:2:30:7:1:true, HEADCROP: 19, LEADING: 15, TRAILING: 15, SLIDINGWINDOW: 4:20, MINLEN: 50) with Trimmomatic v0.39 [29] to remove Nextera adapters and low-quality reads. The trimmed reads were then aligned to the contamination control library (negative control sample) using Bowtie2 v2.5.4 [30] to remove reads derived from laboratory-related contamination. The remaining reads were assembled into contigs using metaSPAdes v4.2.0 [31]. Contigs were subsequently filtered on length (>200 bp) and clustered based on their coverage and identity threshold (80 and 95%, respectively). Taxonomic classification of the contigs was performed using Diamond v2.1.11 [32] against the NCBI nr database and visualized with KronaTools v2.8 [33] using a common ancestor approach. Reads were subsequently mapped to the classified contigs using bwa-mem2 v2.2.1 [34] to quantify their abundance. The contigs corresponding to the Yezo virus genome exhibited average coverage values of 5356×, 575×, and 373× for the L, M, and S segment contigs, respectively. The 3′ end of the detected M-segment sequence lacks 48 nucleotides compared with the reference sequence (NC_079098.1). The 5′ end of the detected S-segment sequence lacks 8 nucleotides, while the 3′ end is missing 55 nucleotides compared with the reference sequence (NC_079100.1).

### 2.4. Sequence Analysis

Sequence analysis and subsequent visualization were performed using Geneious v2025.1.3 software (Biomatters, Auckland, New Zealand) and the ExPASy [35]. The genome organization scheme was constructed based on annotated metagenomic sequences using the online graphical editor BioRender. Protein domain organization was predicted using InterProScan v106.0 [36], and protein transmembrane domains were predicted using DeepTMHMM v1.0.44 [37]. Signal peptide and signal peptidase cleavage site were predicted with ProP v1.0 [38] and SignalP v6.0 [39], respectively. N- and O-glycosylation sites were predicted using NetNGlyc v1.0 [40] and NetOGlyc v4.0 [41], respectively.

### 2.5. Phylogenetic Analysis

For phylogenetic analysis, nucleotide sequences of the L, M, and S segments of YEZV and Sulina virus (SULV) isolates (Table S1), as well as the amino acid sequences of the L segment from representatives of the genus *Orthonairovirus* (Table S2), were used. Multiple sequence alignment was performed using the MAFFT v7.511 online server [42], with the automatic selection of the best alignment algorithm. Phylogenetic trees were constructed using IQ-TREE 3 [43] with 1000 bootstrap replicates, and the most appropriate substitution models were selected using ModelFinder [44]. The selected models were GTR + F + J + G4 for the L segment, TIM2 + F + J + R2 for the M segment, TPM2u + F + J + R2 for the S segment, and Q.YEAST + F + J + R7 for the L segment amino acid sequences. Phylogenetic trees were visualized in R using the ggtree v3.6.2 [45] and phytools v2.4-4 [46] packages. Evolutionary distances of the amino acid and nucleotide sequences for the three YEZV segments were estimated using the p-distance method [47], and the resulting values were converted to identity percentages. The results were further visualized in Python v3.14 using the seaborn v0.13.2 [48] and matplotlib v3.10.3 [49] packages.

## 3. Results

### 3.1. Characterization of the Metagenomic Library

**Figure 1:**
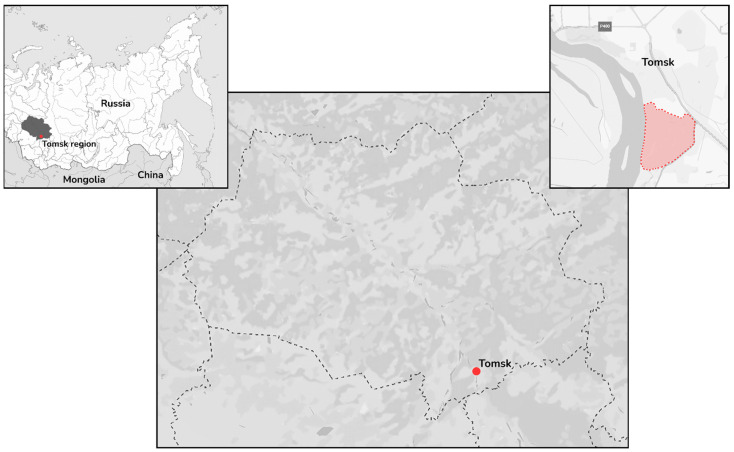
Geographic map of the tick sampling area. Sampling areas are indicated by a red dotted line.

In June 2024, a total of 12 *I. pavlovskyi* ticks were collected from the vegetation in the park area of the Tomsk Polytechnic University “Polytechnic” stadium (Figure 1) and combined into two pools.

After sample preparation according to the modified NetoVIR protocol, the tick samples were combined into a single pool. A cDNA library was then prepared from this pool, followed by metatranscriptomic sequencing, which generated 23,418,766 reads. Analysis of the raw data yielded 196 viral contigs (0.5% of the total). Of these, 93 belonged to bacteriophages (families *Haloferuviridae*, *Shortaselviridae*, *Stanwilliamsviridae*, *Autographviridae*, *Soleiviridae*, *Casjensviridae*, *Straboviridae*, *Zierdtviridae*), 37 to DNA viruses (*Baculoviridae*, *Bamfordviridae*, *Microviridae*, *Genomoviridae*, *Parvoviridae* and *Inoviridae*), 55 to RNA viruses (*Baculoviridae*, *Deltaflexiviridae*, *Virgaviridae*, *Tombusviridae*, *Totiviridae*, *Sedoreoviridae*, *Partitiviridae*, *Hypoviridae*, *Nairoviridae*, *Peribunyaviridae*, *Rhabdoviridae*, *Retroviridae*, *Botourmaviridae*), and 11 to unclassified viruses. The dominant viral taxa are shown in Table 1. For YEZV, four contigs covering >99% of the genome with an average read depth of >1500 were obtained.

### 3.2. Genomic Characterization of the Detected Yezo Virus

**Figure 2:**
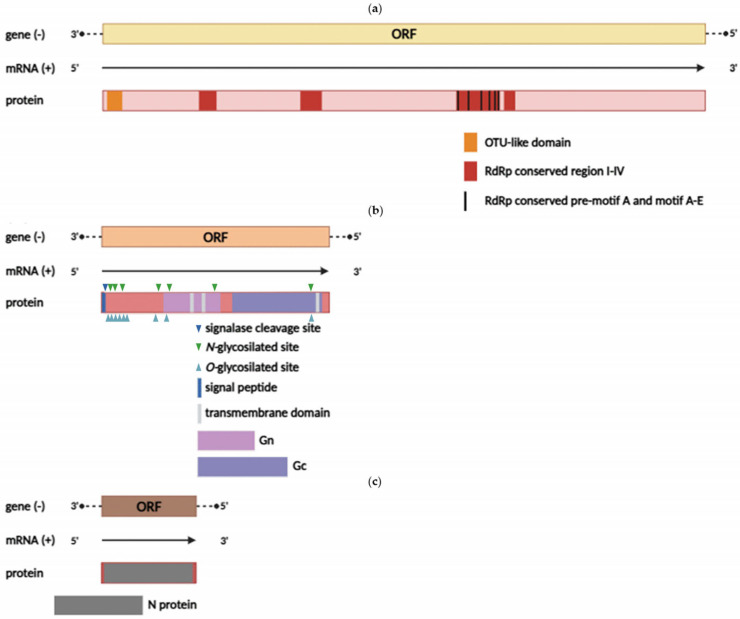
Schematic map of the YEZV genome. (**a**) Segment L and the encoded RdRp; (**b**) segment M and the encoded GPC; (**c**) segment S and the encoded nucleoprotein.

The YEZV genome is a linear ssRNA (–) consisting of three segments (Figure 2).

The L segment is 12,103 nt in length and encodes an RNA-dependent RNA polymerase (RdRp) (3938 aa). Previous studies have shown that RdRp contains an OTU-like protease domain, a polymerase module consisting of pre-motif A and motifs A–E, and four conserved regions (I–IV) [50,51]. Amino acid sequence alignment confirmed that the YEZV RdRp contains all the highly conserved regions (Figure S1). Additionally, 45 amino acid substitutions were identified in the RdRp protein structure, including 18 radical amino acid replacements (Table S3), which may affect protein structure or function.

The M segment is 4247 nt in length and encodes a glycoprotein precursor complex (GPC) (1356 aa), which consists of two subunits—Gn (from 369 to 687 aa) and Gc (from 777 to 1319 aa). The N-terminal signal peptide and its cleavage site were predicted between residues 21 and 22 aa. The N-terminal portion of the glycoprotein contains a high density of O- and N-glycosylation sites and is susceptible to proteolytic cleavage. The Gn protein includes one O- and two N-glycosylation sites, two transmembrane domains (541–560 aa and 668–682 aa), and a pair of conserved zinc finger domains, which are also present in Meihua Mountain virus and Crimean-Congo hemorrhagic fever viruses (CCHFV) [51,52]. The Gc protein contains one O- and one N-glycosylation site and one transmembrane domain located between 1283 and 1303 aa. In addition, the nucleoprotein of the YEZV has conserved fusion loop regions (bc loop, cd loop, ij loop), like those observed in other orthonairoviruses [53] (Figure S2). It was also found that the glycoprotein complex has 80 amino acid substitutions, including 32 radical amino acid replacements (Table S4).

The S segment is 1685 nt in length and encodes a nucleocapsid protein (502 aa). Structural studies of nairovirus nucleocapsid proteins have revealed two main domains: a globular head and a stem, along with sites that are necessary for RNA/DNA binding [54]. The alignment of the nucleotide sequences of the two domains showed that YEZV isolates differ from viruses of the NSD (Nairobi sheep disease) genogroup while exhibiting high conservation among YEZV isolates (Figure S3). Additionally, 16 amino acid substitutions were identified in nucleoprotein structure, including 9 radical amino acid replacements (Table S5).

### 3.3. Nucleotide and Amino Acid Identity

To assess the nucleotide (Figure S4) and amino acid (Figure S5) identity of the genome segments of the identified YEZV, comparative analysis was performed with genome segments sequences from other virus isolates. The nucleotide identity of the L segment ranged from 90.848% to 91.399%, with the highest value observed for isolate PV061572.1 (*I. persulcatus*, Khabarovsk region, Far East). For the M segment, identity values ranged from 89.336% to 90.054%, with the closest match being isolate PQ475627.1 (*I. persulcatus*, Inner Mongolia, China). For the S segment, identity values ranged from 91.328% and 92.341%, with the highest identity observed for isolate PV061580.1 (*I. persulcatus*, Primorsky region, Far East).

Amino acid identity for the RdRp protein ranged from 98.502% to 98.959%, with the highest value recorded for isolate WWT48702.1 (*I. persulcatus*, Jilin Province, China). According to the ICTV species demarcation criteria (<93% identity in the L amino acid sequence for a new species within the genus *Orthonairovirus*), the detected virus belongs to YEZV. For the GPC, amino acid identity ranged from 93.732% to 94.764%, with the closest match being isolate XJP49230.1 (*I. persulcatus*, Inner Mongolia, China). For the nucleoprotein, identity values ranged from 96.414% to 97.200%, with the highest identity observed for isolate XJQ61045.1 (*Homo sapiens* (human metagenome), Heilongjiang Province, China).

### 3.4. Phylogenetic Analysis

**Figure 3:**
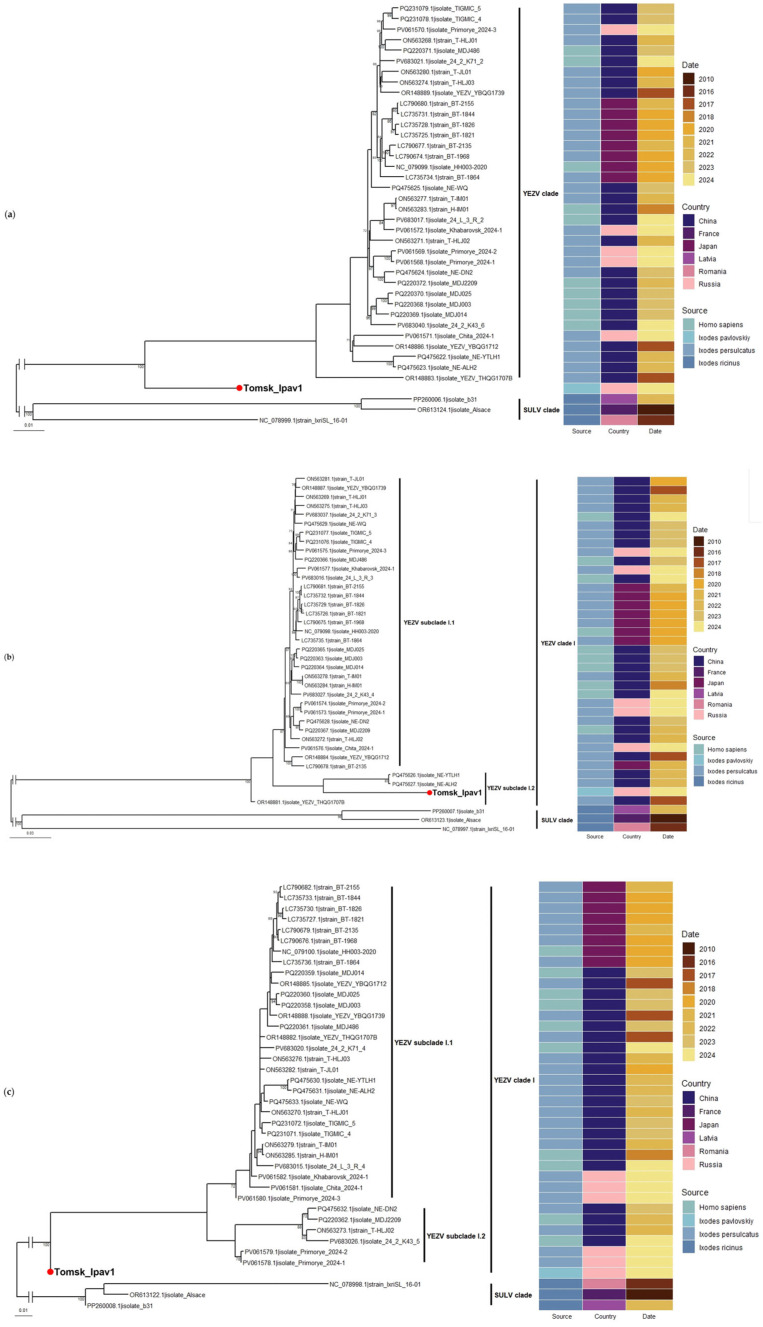
Maximum likelihood phylogenetic tree based on the nucleotide sequences of YEZV and SULV isolates. (**a**) Segment L; (**b**) segment M; (**c**) segment S. The red dot indicates the sequences of YEZV from the study. Bootstrap values ≥ 70 are indicated.

Phylogenetic analysis based on the nucleotide sequences of the three YEZV genome segments (L, M, S) demonstrated that all isolates of this virus form a well-supported monophyletic clade, clearly distinct from the closely related SULV. The isolates showed no specific clustering pattern based on the source and year of virus isolation. Our newly identified YEZV isolate (Tomsk_Ipav1) formed a sister clade to previously described virus isolates based on the L and S segments. For the M segment, our isolate exhibited a closer evolutionary relationship with two Chinese isolates (Figure 3). However, bootstrap support for this grouping was low.

**Figure 4:**
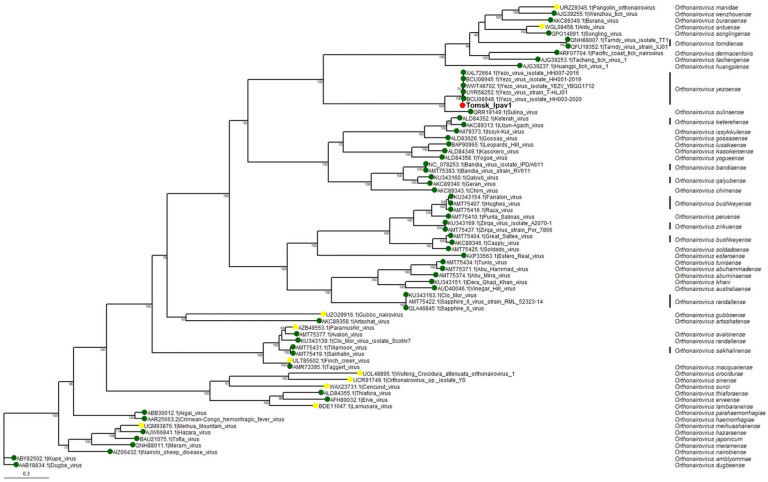
Maximum likelihood phylogenetic tree of the amino acid sequences of the L segment of *Orthonairovirus.* The red dot indicates the sequence of YEZV from the study. The green dots indicate the sequence of member species. The yellow dots indicate the sequence of related and unclassified species. Bootstrap support values ≥ 70 are indicated.

To clarify the evolutionary relationships of the new isolate within the genus *Orthonairovirus*, a phylogenetic tree was constructed based on the amino acid sequence of RdRp (Figure 4).

The analysis included 65 representatives of the genus *Orthonairovirus* and 6 YEZV isolates, including the newly identified isolate. The resulting phylogram shows that all YEZV isolates, including the variant detected in the study, form a well-supported species subclade, clearly distinct from other viruses in the genus. Overall, the tree structure is consistent with previously published data [21]. Thus, the relatively high percentage of differences observed in the virus genome segments, based on amino acid and nucleotide sequences comparisons among YEZV isolates, as well as the phylogenetic analysis, indicate the presence of a new genetic variant of Yezo virus circulating in the Tomsk region (Western Siberia, Russia).

## 4. Discussion

In the present study, we provide the first evidence of YEZV in the Tomsk region (Western Siberia), significantly expanding its geographical range beyond the previously reported regions of Japan, China, and Russia. In Russia, the virus had previously been detected only in Eastern Siberia (Zabaykalsky region) and the Far East (Primorsky and Khabarovsk regions), with no YEZV detected among 952 samples from Western Siberia, including the Altai Republic (n = 79), Kemerovo region (n = 289), Novosibirsk region (n = 107), Tomsk region (n = 340) and Tyumen region (n = 137) [10]. Our analysis revealed that the oligonucleotides used in previous study contain single-nucleotide mismatches, which may reduce PCR sensitivity or even prevent detection of the YEZV genetic variant identified in this study. Another important finding was the detection of the virus in the ixodid tick *I. pavlovskyi*, whereas previous studies in Russia reported detections exclusively in *I. persulcatus* (with *I. persulcatus* and *I. ricinus* being studied). Data from other countries indicate that various ixodid tick species, including *D. silvarum*, *H. megaspinosa*, *H. japonica*, *I. ovatus* and *I. persulcatus*, can also carry this virus [10,13,14,15,16,17,18,19].

As a result of metagenomic sequencing, we obtained the complete sequences of all three genome segments of YEZV, with high-quality allowing detailed genomic characterization. Specifically, the L segment encodes an RdRp and includes an OTU-like protease domain and a polymerase module consisting of pre-motif A and motifs A–E, and four conserved regions (I–IV). The OTU-like protease domain belongs to a group of deubiquitinases that disrupt the innate immune response and enhance viral infectivity, as shown for CCHFV, by modifying the antiviral type I interferon response. It may therefore represent a promising drug target [55].

The M segment encodes a GPC that consists of two subunits, Gn and Gc. In addition, the GPC contains an N-terminal signal peptide with its cleavage site, O- and N-glycosylation rich regions, a pair of conserved zinc finger domains, and conserved fusion loop regions—bc, cd, and ij loops. The presence of two zinc finger domains in the Gn tail region of the Yezo virus probably indicates their potential role in RNA interaction and virus assembly, as demonstrated for other orthonairoviruses (CCHFV, Dugbe and Kupe virus) [52,56]. In addition, conserved fusion loop regions that form part of the Gc protein play a critical role in viral and host cell membrane fusion [53].

The S segment encodes a nucleoprotein consisting of two domains: a globular “head” and “stalk”. These domains are important for viral RNA binding and oligomerization, facilitating the formation of ribonucleoprotein complexes [10]. All of these structural components are consistent with those found in other representatives of the genus *Orthonairovirus* [10,50,51,52,53,54]. Amino acid substitutions were observed in all of the proteins analyzed, with some resulting in a change in amino acid class, potentially altering protein’s conformation and function. The greatest number of substitutions was observed in the glycoprotein, which may reflect viral adaptation to a relatively new vector, the *I. pavlovskyi* tick.

Analysis of nucleotide and amino acid sequence identity demonstrated that our YEZV isolate is similar to previously published sequences, but the relatively high level of divergences suggests the discovery of a new genetic variant. Nucleotide differences ranged from 8.601% to 9.152% for the L segment, 9.946% to 10.664% for the M segment, and 7.659% to 8.672% for the S segment. At the protein level, differences ranged from 1.041% to 1.498% for the RdRp, 5.236% to 6.268% for the GPC, and 2.8% to 3.586% for the nucleoprotein. These findings confirm previous observations that the M segment accumulates the most mutations [57]. The increased level of genetic variability in the M segment compared to the other two genome segments is likely due to its functional significance, which lies in encoding the glycoproteins located on the surface of the viral particle. Since these proteins are responsible for viral attachment and entry into the cell, they are subject to selective pressure from the host’s immune system and during adaptation to different host and vector species. This ensures the virus’s adaptability to new conditions.

Phylogenetic analysis based on the nucleotide sequences from all three genome segments and the RdRp amino acid sequence confirmed the affiliation of the new genetic variant within the YEZV clade. For the L and S segments, the genetic variant forms a distinct, well-supported sister branch relative to other virus isolates, highlighting its genetic distinctiveness, which may relate to its discovery in a different ixodid tick species and circulation in a geographically remote region. Meanwhile, for the M segment, the new genovariant shows a closer relationship with two Chinese isolates.

The discrepancies in phylogenetic tree topology indicate differences in the evolutionary dynamics among genome segments, likely due to reassortment. Sequence identity data support this hypothesis: the L segment is most similar to the isolate from the Khabarovsk region, the M segment to the isolate from Inner Mongolia, and the S segment to the isolate from the Primorsky region. Similar patterns were observed at the protein level: RdRp is most similar to the isolate from Jilin Province, GPC to the isolate from Inner Mongolia, and nucleoprotein to the isolate from Heilongjiang Province. Reassortment events have previously been reported in other orthonairoviruses, including Uzhun-Agach virus, CCHFV, and Paramushir virus [58,59,60].

From an epidemiological perspective, *I. pavlovskyi* is the predominant ixodid tick species in urban biotopes of Tomsk, exhibiting strong ornithophilicity at all developmental stages [11,12]. Detection of a new YEZV genovariant in this species may result from frequent contact with birds, whose migration facilitates cross-border pathogen transfer. As the YEZV has been detected in animals and ixodid ticks in Japan and China [10,13,14,15,16,17,18,19], it is possible that the virus is circulating among vertebrates in Western Siberia, although no data currently to confirm this.

Thus, our findings expand knowledge of the geographic distribution, genome size and structural organization of YEZV, highlighting the need for ongoing epidemiological surveillance of the pathogen. Although the pathogenic potential of the Yezo virus in humans and animals remains unclear, its presence in ticks, animals and reported human cases suggests a potential risk for human health. Future research should focus on expanding the collection sites, developing sensitive RT-PCR assays for detecting new and known variants, assessing virus prevalence among other relevant ixodid tick species in Western Siberia and evaluating vertebrate host susceptibility to better define the virus’s host range.

## 5. Conclusions

The YEZV, due to its clinical manifestation, represents a significant public health concern. In this study, we report the first detection of YEZV in Western Siberia, substantially expanding its geographical range. Our findings also document the virus in *I. pavlovskyi* for the first time, thereby extending the range of its potential vectors. Using metagenomic sequencing, we obtained and characterized the complete sequences of the three YEZV genome segments. Our analysis revealed that the isolate represents a novel genetic variant, as demonstrated by sequence identity results and phylogenetic analysis. The highest number of mutations was found in the M segment, indicating its key role in adapting to new hosts and vectors. Differences in phylogenetic tree topology among the three segments suggest the possibility of reassortment. This highlights the complex evolutionary dynamics of the virus and explains its genetic diversity. These results expand our understanding of the virus’s distribution and its vectors, emphasizing the importance of further epidemiological surveillance.

## Figures and Tables

**Table 1:** Composition of dominant viral taxa identified in the sample, based on average logarithm of the e-value.

Taxon (Closest BLASTx Match)	Count of Contigs	Relative Abundance (%)	Coverage of Contigs	Length of Contigs (nt)
YEZV (*Nairoviridae*) (94.91–98.76%)	4	66	1, 373, 575 and 5356	252, 1748, 4296 and 12,103
Fangzheng tomsu-like virus (*Tombusviridae*) (94.24–96.11%)	3	28	920, 3132 and 6339	3211, 1094 and 1479
Great Island virus (*Sedoreoviridae*) (28.47% and 32.51%)	2	3	615 and 904	3031 and 2165
Haerbin Reovi tick virus 1 (Unclassified *Reiovirales*) (99.46%)	1	2	690	4149

## Data Availability

Partial genome sequences of YEZV from *I. pavlovskyi* were deposited in GenBank (PX219864, PX219865, PX219866). Raw sequencing reads are available under accession number SRR35181648.

## Supplementary Materials

The following supporting information can be downloaded at: https://www.mdpi.com/article/10.3390/v17101362/s1, Table S1: Viruses and GenBank accession numbers of nucleotide (nt) and amino acid (aa) sequences of YEZV and nucleic acid sequences of SULV isolates used in phylogenetic and other comparative analyses; Table S2: Viruses and GenBank accession numbers of amino acid sequences of RdRp of members of the genus *Orthonairovirus* used in phylogenetic analyses; Table S3: Amino acid substitutions in the RNA-dependent RNA polymerase (L segment) of the new YEZV genetic variant compared to Japanese and Russian YEZV isolates; Table S4: Amino acid substitutions in the glycoprotein complex (M segment) of the new YEZV genetic variant compared to Japanese and Russian YEZV isolates; Table S5: Amino acid substitutions in the nucleoprotein (S segment) of the new YEZV genetic variant compared to Japanese and Russian YEZV isolates; Figure S1: Amino acid sequence alignment of the conserved regions of NSD viruses and YEZV isolates; Figure S2: Amino acid sequence alignment of the Gns and Gcs of NSD genogroup viruses and YEZV isolates; Figure S3: Amino acid sequence alignment of the nucleoproteins of NSD genogroup viruses and YEZV isolates; Figure S4: Pairwise nucleic acid identity matrix of YEZV isolates; Figure S5: Pairwise amino acid identity matrix of YEZV isolates.

## Abbreviations

The following abbreviations are used in this manuscript:

YEZV	Yezo virus
TBEV	Tick-borne encephalitis virus
ssRNA (–)	Single-stranded negative-sense RNA
SULV	Sulina virus
RdRp	RNA-dependent RNA polymerase
GPC	Glycoprotein precursor complex
CCHFV	Crimean-Congo hemorrhagic fever virus
NSD	Nairobi sheep disease

## References

[1] Latif A.A., Putterill J.F., De Klerk D.G., Pienaar R., Mans B.J. (2012). *Nuttalliella Namaqua* (Ixodoidea: Nuttalliellidae): First description of the male, immature stages and re-description of the female. PLoS ONE.

[2] Maqbool M., Sajid M.S., Saqib M., Anjum F.R., Tayyab M.H., Rizwan H.M., Rashid M.I., Rashid I., Iqbal A., Siddique R.M. (2022). Potential mechanisms of transmission of tick-borne viruses at the virus-tick interface. Front. Microbiol..

[3] Moskvitina N.S., Romanenko V.N., Ternovoi V.A., Ivanova N.V., Protopopova E.V., Kravchenko L.B., Kononova Y.V., Kuranova V.N., Chausov E.A., Moskvitin S.S. (2008). Detection of the West Nile virus and its genetic typing in ixodid ticks (Parasitiformes: Ixodidae) in Tomsk city and its suburbs. Parazitologiya.

[4] Kuranova V.N., Yartsev V.V., Kononova Y.V., Protopopova E.V., Konovalova S.N., Ternovoi V.A., Tavkina I.S., Romanenko V.N., Loktev V.B., Moskvitina N.S. (2009). Lacertids (Sauria, Lacertidae) in natural foci of infectious human-transmitted ecosystems of the south-east territories of Western Siberia. Proceedings of the 4th Meeting of the Nikolsky Herpetological Society.

[5] Vilibić-Čavlek T., Bogdanić M., Savić V., Barbić L., Stevanović V., Kaić B. (2024). Tick-Borne Human Diseases Around the Globe.

[6] Romanenko V.N. (2005). The Peculiarities of the biology of ticks inhabiting the environs of Tomsk city. Parazitologiya.

[7] Korobitsyn I.G., Moskvitina N.S., Tyutenkov O.Y., Gashkov S.I., Kononova Y.V., Moskvitin S.S., Romanenko V.N., Mikryukova T.P., Protopopova E.V., Kartashov M.Y. (2021). Detection of tick-borne pathogens in wild birds and their ticks in Western Siberia and high level of their mismatch. Folia Parasitol..

[8] Voronkova O.V., Romanenko V.N., Simakova A.V., Esimova I.E., D’yakov D.A., Motlokhova E.A., Chernyshov N.A., Yamaletdinova D.M. (2023). Analysis of multiple infection in ixodid ticks *Dermacentor reticulatus* in a combined natural focus of vector-borne infections in the Tomsk region. Probl. Osob. Opasnykh Infektsii.

[9] Tupota N.L., Ternovoi V.A., Ponomareva E.P., Bayandin R.B., Shvalov A.N., Malyshev B.S., Tregubchak T.V., Bauer T.V., Protopopova E.V., Petrova N.K. (2023). Detection of the genetic material of the viruses Tacheng uukuvirus and Sara tick phlebovirus in taiga ticks collected in the Sverdlovsk, Tomsk regions and Primorsky territory of Russia and their phylogeny. Probl. Osob. Opasnykh Infektsii.

[10] Kartashov M., Svirin K., Zheleznova A., Yanshin A., Radchenko N., Kurushina V., Tregubchak T., Maksimenko L., Sivay M., Ternovoi V. (2025). A first report of the Yezo virus isolates detection in Russia. Viruses.

[11] Romanenko V.N. (2011). Long-term dynamics of density and diversity of ticks (Ixodidae) on the natural and disturbed territories. Parazitologiya.

[12] Moskvitina N.S., Korobitsyn I.G., Tyuten’kov O.Y., Gashkov S.I., Kononova Y.V., Moskvitin S.S., Romanenko V.N., Mikryukova T.P., Protopopova E.V., Kartashov M.Y. (2014). The role of birds in the maintenance of tick-borne infections in the Tomsk anthropurgic foci. Biol. Bull. Russ. Acad. Sci..

[13] Kodama F., Yamaguchi H., Park E., Tatemoto K., Sashika M., Nakao R., Terauchi Y., Mizuma K., Orba Y., Kariwa H. (2021). A novel nairovirus associated with acute febrile illness in Hokkaido, Japan. Nat. Commun..

[14] Lv X., Liu Z., Li L., Xu W., Yuan Y., Liang X., Zhang L., Wei Z., Sui L., Zhao Y. (2023). Yezo virus infection in tick-bitten patient and ticks, Northeastern China. Emerg. Infect. Dis..

[15] Ni X.-B., Cui X.-M., Liu J.-Y., Ye R.-Z., Wu Y.-Q., Jiang J.-F., Sun Y., Wang Q., Shum M.H.-H., Chang Q.-C. (2023). Metavirome of 31 tick species provides a compendium of 1,801 RNA virus genomes. Nat. Microbiol..

[16] Kumar P., Priyanshu P., Sharma R.K., Sharma D., Arora M., Gaidhane A.M., Zahiruddin Q.S., Rustagi S., Mawejje E., Satapathy P. (2024). Climate change and its role in the emergence of new tick-borne Yezo virus. New Microbes New Infect..

[17] Nishino A., Tatemoto K., Ishijima K., Inoue Y., Park E., Yamamoto T., Taira M., Kuroda Y., Virhuez-Mendoza M., Harada M. (2024). Transboundary movement of Yezo virus via ticks on migratory birds, Japan, 2020–2021. Emerg. Infect. Dis..

[18] Ogata Y., Sato T., Kato K., Kikuchi K., Mitsuhashi K., Watari K., Tamiya K., Goto A., Yamaguchi H., Hisada R. (2024). A Case of tick-borne Yezo virus infection: Concurrent detection in the patient and tick. Int. J. Infect. Dis..

[19] Suzuki K., Suzuki S., Yamaguchi H., Kakinoki Y. (2024). Tick-borne disease with Yezo virus and *Borrelia miyamotoi* coinfection. Intern. Med..

[20] Matsuno K. (2025). Endemic area of emerging tick-borne Yezo virus infections discovered in China. Lancet Infect. Dis..

[21] Kuhn J.H., Alkhovsky S.V., Avšič-Županc T., Bergeron É., Burt F., Ergünay K., Garrison A.R., Marklewitz M., Mirazimi A., Papa A. (2024). ICTV virus taxonomy profile: *Nairoviridae* 2024: This Article Is Part of the ICTV Virus Taxonomy Profiles Collection. J. Gen. Virol..

[22] Kuhn J., Wiley M., Rodriguez S., Bào Y., Prieto K., Travassos Da Rosa A., Guzman H., Savji N., Ladner J., Tesh R. (2016). Genomic characterization of the genus *Nairovirus* (family *Bunyaviridae*). Viruses.

[23] Spengler J.R., Estrada-Peña A., Garrison A.R., Schmaljohn C., Spiropoulou C.F., Bergeron É., Bente D.A. (2016). A Chronological review of experimental infection studies of the role of wild animals and livestock in the maintenance and transmission of Crimean-Congo hemorrhagic fever virus. Antivir. Res..

[24] Zhao Y., Zhang W., Zhang X. (2024). Application of metagenomic next-generation sequencing in the diagnosis of infectious diseases. Front. Cell. Infect. Microbiol..

[25] Vandegrift K.J., Kapoor A. (2019). The ecology of new constituents of the tick virome and their relevance to public health. Viruses.

[26] Filippova N.A., Strelkov A.A. (1977). Ixodid Ticks of the Subfamily Ixodinae.

[27] Conceição-Neto N., Yinda K.C., Van Ranst M., Matthijnssens J., Moya A., Pérez Brocal V. (2018). NetoVIR: Modular Approach to Customize Sample Preparation Procedures for Viral Metagenomics.

[28] De Coninck L., Faye L., Basler N., Jansen D., Van Espen L. (2025). ViPER, Version 2.3.1.

[29] Bolger A.M., Lohse M., Usadel B. (2014). Trimmomatic: A flexible trimmer for Illumina sequence data. Bioinformatics.

[30] Langmead B., Salzberg S.L. (2012). Fast gapped-read alignment with Bowtie 2. Nat. Methods.

[31] Nurk S., Meleshko D., Korobeynikov A., Pevzner P.A. (2017). metaSPAdes: A new versatile metagenomic assembler. Genome Res..

[32] Buchfink B., Xie C., Huson D.H. (2015). Fast and sensitive protein alignment using DIAMOND. Nat. Methods.

[33] Ondov B.D., Bergman N.H., Phillippy A.M. (2011). Interactive metagenomic visualization in a web browser. BMC Bioinform..

[34] Vasimuddin M.D., Misra S., Li H., Aluru S. (2019). Efficient architecture-aware acceleration of BWA-MEM for multicore systems. Proceedings of the 2019 IEEE International Parallel and Distributed Processing Symposium (IPDPS).

[35] Gasteiger E. (2003). ExPASy: The proteomics server for in-depth protein knowledge and analysis. Nucleic Acids Res..

[36] Jones P., Binns D., Chang H.-Y., Fraser M., Li W., McAnulla C., McWilliam H., Maslen J., Mitchell A., Nuka G. (2014). InterProScan 5: Genome-scale protein function classification. Bioinformatics.

[37] Hallgren J., Tsirigos K.D., Pedersen M.D., Almagro Armenteros J.J., Marcatili P., Nielsen H., Krogh A., Winther O. (2022). DeepTMHMM predicts alpha and beta transmembrane proteins using deep neural networks. Bioinformatics.

[38] Duckert P., Brunak S., Blom N. (2004). Prediction of proprotein convertase cleavage sites. Protein Eng. Des. Sel..

[39] Teufel F., Almagro Armenteros J.J., Johansen A.R., Gíslason M.H., Pihl S.I., Tsirigos K.D., Winther O., Brunak S., Von Heijne G., Nielsen H. (2022). SignalP 6.0 Predicts all five types of signal peptides using protein language models. Nat. Biotechnol..

[40] Gupta R., Brunak S. (2002). Prediction of glycosylation across the human proteome and the correlation to protein function. Pac. Symp. Biocomput..

[41] Steentoft C., Vakhrushev S.Y., Joshi H.J., Kong Y., Vester-Christensen M.B., Schjoldager K.T.-B.G., Lavrsen K., Dabelsteen S., Pedersen N.B., Marcos-Silva L. (2013). Precision mapping of the human O-GalNAc glycoproteome through SimpleCell technology. EMBO J..

[42] Katoh K., Rozewicki J., Yamada K.D. (2019). MAFFT online service: Multiple sequence alignment, interactive sequence choice and visualization. Brief. Bioinform..

[43] Wong T., Ly-Trong N., Ren H., Baños H., Roger A., Susko E., Bielow C., De Maio N., Goldman N., Hahn M. (2025). IQ-TREE 3: Phylogenomic inference software using complex evolutionary models. Life Sci..

[44] Kalyaanamoorthy S., Minh B.Q., Wong T.K.F., Von Haeseler A., Jermiin L.S. (2017). ModelFinder: Fast model selection for accurate phylogenetic estimates. Nat. Methods.

[45] Yu G., Smith D.K., Zhu H., Guan Y., Lam T.T. (2017). Ggtree: An r package for visualization and annotation of phylogenetic trees with their covariates and other associated data. Methods Ecol. Evol..

[46] Revell L.J. (2012). Phytools: An R package for phylogenetic comparative biology (and other things). Methods Ecol. Evol..

[47] Tamura K., Stecher G., Kumar S. (2021). MEGA11: Molecular evolutionary genetics analysis version 11. Mol. Biol. Evol..

[48] Waskom M. (2021). Seaborn: Statistical data visualization. JOSS.

[49] Hunter J.D. (2007). Matplotlib: A 2D graphics environment. Comput. Sci. Eng..

[50] Honig J.E., Osborne J.C., Nichol S.T. (2004). Crimean–Congo hemorrhagic fever virus genome L RNA segment and encoded protein. Virology.

[51] Zhang X., Li H.-Y., Shao J.-W., Pei M.-C., Cao C., Huang F.-Q., Sun M.-F. (2022). Genomic characterization and phylogenetic analysis of a novel Nairobi sheep disease genogroup *Orthonairovirus* from ticks, Southeastern China. Front. Microbiol..

[52] Estrada D.F., De Guzman R.N. (2011). Structural characterization of the Crimean-Congo hemorrhagic fever virus Gn tail provides insight into virus assembly. J. Biol. Chem..

[53] Li N., Rao G., Li Z., Yin J., Chong T., Tian K., Fu Y., Cao S. (2022). Cryo-EM structure of glycoprotein C from Crimean-Congo hemorrhagic fever virus. Virol. Sin..

[54] Wang W., Liu X., Wang X., Dong H., Ma C., Wang J., Liu B., Mao Y., Wang Y., Li T. (2015). Structural and functional diversity of Nairovirus-encoded nucleoproteins. J. Virol..

[55] Chen Z.-Y., Zhang J., He P.-J., Xiong T., Zhu D.-Y., Zhu W.-J., Ni X.-B., Du L.-F., Wang Q., Zhang Y.-W. (2025). Characteristics of viral ovarian tumor domain protease from two emerging orthonairoviruses and identification of Yezo virus human infections in northeastern China as early as 2012. J. Virol..

[56] Ndiaye M., Badji A., Dieng I., Dolgova A.S., Mhamadi M., Kirichenko A.D., Gladkikh A.S., Gaye A., Faye O., Sall A.A. (2024). Molecular detection and genetic characterization of two Dugbe orthonairovirus isolates detected from ticks in southern Senegal. Viruses.

[57] Deyde V.M., Khristova M.L., Rollin P.E., Ksiazek T.G., Nichol S.T. (2006). Crimean-Congo hemorrhagic fever virus genomics and global diversity. J. Virol..

[58] Al’khovskiĭ S.V., L’vov D.K., Shchelkanov M.I., Deriabin P.G., Shchetinin A.M., Samokhvalov E.I., Aristova V.A., Gitel’man A.K., Botikov A.G. (2014). Genetic characterization of the Uzun-Agach virus (UZAV, *Bunyaviridae*, *Nairovirus*), isolated from bat *Myotis blythii oxygnathus* Monticelli, 1885 (Chiroptera; Vespertilionidae) in Kazakhstan. Vopr. Virusol..

[59] Lukashev A.N., Klimentov A.S., Smirnova S.E., Dzagurova T.K., Drexler J.F., Gmyl A.P. (2016). Phylogeography of Crimean Congo hemorrhagic fever virus. PLoS ONE.

[60] Safonova M.V., Shchelkanov M.Y., Khafizov K., Matsvay A.D., Ayginin A.A., Dolgova A.S., Shchelkanov E.M., Pimkina E.V., Speranskaya A.S., Galkina I.V. (2019). Sequencing and genetic characterization of two strains Paramushir virus obtained from the Tyuleniy Island in the Okhotsk Sea (2015). Ticks Tick. Borne Dis..

