# Supplementary material for: Genomic Characterization of a Novel Yezo Virus Revealed in Ixodes pavlovskyi Tick Virome in Western Siberia
Source: Viruses. 2025 Oct 11;17(10):1362. doi: 10.3390/v17101362 (PMC12567925; doi:10.3390/v17101362)
Supplement: Supplementary file 1 [file viruses-17-01362-s001.zip › viruses-3882135-supplementary.pdf]

Table S1. Viruses and GenBank accession numbers of nucleotide (nt) and amino acid (aa) sequences of YEZV and nucleic acid sequences of SULV isolates used in phylogenetic and other comparative analyses.

Virus (strain or isolate)	Segment	GenBank accession number nt/aa
Yezo virus (24 2 K43 6)	L	PV683040.1 / XUJ59931.1
Yezo virus (24 2 K43 4)	M	PV683027.1 / XUJ59927.1
Yezo virus (24 2 K43 5)	S	PV683026.1 / XUJ59925.1
Yezo virus (24 2 K71 2)	L	PV683021.1 / XUJ59930.1
Yezo virus (24 2 K71 3)	M	PV683037.1 / XUJ59928.1
Yezo virus (24 2 K71 4)	S	PV683020.1 / XUJ59924.1
Yezo virus (24 L 3 R 2)	L	PV683017.1 / XUJ59929.1
Yezo virus (24 L 3 R 3)	M	PV683016.1 / XUJ59926.1
Yezo virus (24 L3 R 4)	S	PV683015.1 / XUJ59923.1
Yezo virus (Khabarovsk 2024-1)	L	PV061572.1 / XPK55866.1
Yezo virus (Khabarovsk 2024-1)	M	PV061577.1 / XPK55871.1
Yezo virus (Khabarovsk 2024-1)	S	PV061582.1 / XPK55876.1
Yezo virus (Chita 2024-1)	L	PV061571.1 / XPK55865.1
Yezo virus (Chita 2024-1)	M	PV061576.1 / XPK55870.1
Yezo virus (Chita 2024-1)	S	PV061581.1 / XPK55875.1
Yezo virus (Primorye 2024-3)	L	PV061570.1 / XPK55864.1
Yezo virus (Primorye 2024-3)	M	PV061575.1 / XPK55869.1
Yezo virus (Primorye 2024-3)	S	PV061580.1 / XPK55874.1
Yezo virus (Primorye 2024-2)	L	PV061569.1 / XPK55863.1
Yezo virus (Primorye 2024-2)	M	PV061574.1 / XPK55868.1
Yezo virus (Primorye 2024-2)	S	PV061579.1 / XPK55873.1
Yezo virus (Primorye 2024-1)	L	PV061568.1 / XPK55862.1
Yezo virus (Primorye 2024-1)	M	PV061573.1 / XPK55867.1
Yezo virus (Primorye 2024-1)	S	PV061578.1 / XPK55872.1
Yezo virus (NE-WQ)	L	PQ475625.1 / XJP49228.1
Yezo virus (NE-WQ)	M	PQ485629.1 / XJP49232.1
Yezo virus (NE-WQ)	S	PQ475633.1 / XJP49236.1
Yezo virus (NE-DN2)	L	PQ475624.1 / XJP49227.1
Yezo virus (NE-DN2)	M	PQ475628.1 / XJP49231.1
Yezo virus (NE-DN2)	S	PQ475632.1 / XJP49235.1
Yezo virus (NE-ALH2)	L	PQ475623.1 / XJP49226.1
Yezo virus (NE-ALH2)	M	PQ475627.1 / XJP49230.1
Yezo virus (NE-ALH2)	S	PQ475631.1 / XJP49234.1
Yezo virus (NE-YTLH1)	L	PQ475622.1 / XJP49225.1
Yezo virus (NE-YTLH1)	M	PQ475626.1 / XJP49229.1
Yezo virus (NE-YTLH1)	S	PQ475630.1 / XJP49233.1
Yezo virus (TIGMIC 5)	L	PQ231079.1 / XJO53607.1
Yezo virus (TIGMIC 5)	M	PQ231077.1 / XJO53605.1
Yezo virus (TIGMIC 5)	S	PQ231072.1 / XJO53600.1
Yezo virus (TIGMIC 4)	L	PQ231078.1 / XJO53606.1
Yezo virus (TIGMIC 4)	M	PQ231076.1 / XJO53604.1
Yezo virus (TIGMIC 4)	S	PQ231071.1 / XJO53599.1
Yezo virus (MDJ2209)	L	PQ220372.1 / XJQ61057.1
Yezo virus (MDJ2209)	M	PQ220367.1 / XJQ61052.1
Yezo virus (MDJ2209)	S	PQ220362.1 / XJQ61047.1
Yezo virus (MDJ486)	L	PQ220371.1 / XJQ61056.1
Yezo virus (MDJ486)	M	PQ220366.1 / XJQ61051.1
Yezo virus (MDJ486)	S	PQ220361.1 / XJQ61047.1

Yezo virus (MDJ025)	L	PQ220370.1 / XJQ61055.1
Yezo virus (MDJ025)	M	PQ220365.1 / XJQ61050.1
Yezo virus (MDJ025)	S	PQ220360.1 / XJQ61045.1
Yezo virus (MDJ014)	L	PQ220369.1 / XJQ61054.1
Yezo virus (MDJ014)	M	PQ220364.1 / XJQ61049.1
Yezo virus (MDJ014)	S	PQ220359.1 / XJQ61044.1
Yezo virus (MDJ003)	L	PQ220368.1 / XJQ61053.1
Yezo virus (MDJ003)	M	PQ220363.1 / XJQ61048.1
Yezo virus (MDJ003)	S	PQ220358.1 / XJQ61043.1
Yezo virus (YEZV YBQG1739)	L	OR148889.1 / WWT48703.1
Yezo virus (YEZV YBQG1739)	M	OR148887.1 / WWT48697.1
Yezo virus (YEZV YBQG1739)	S	OR148888.1 / WWT48700.1
Yezo virus (YEZV YBQG1712)	L	OR148886.1 / WWT48702.1
Yezo virus (YEZV YBQG1712)	M	OR148884.1 / WWT48696.1
Yezo virus (YEZV YBQG1712)	S	OR148885.1 / WWT48699.1
Yezo virus (YEZV THQG1707B)	L	OR148883.1 / WWT48701.1
Yezo virus (YEZV THQG1707B)	M	OR148881.1 / WWT48695.1
Yezo virus (YEZV THQG1707B)	S	OR148882.1 / WWT48698.1
Yezo virus (H-IM01)	L	ON563283.1 / UYR58267.1
Yezo virus (H-IM01)	M	ON563284.1 / UYR58268.1
Yezo virus (H-IM01)	S	ON563285.1 / UYR58269.1
Yezo virus (T-JL01)	L	ON563280.1 / UYR58264.1
Yezo virus (T-JL01)	M	ON563281.1 / UYR58265.1
Yezo virus (T-JL01)	S	ON563282.1 / UYR58266.1
Yezo virus (T-IM01)	L	ON563277.1 / UYR58261.1
Yezo virus (T-IM01)	M	ON563278.1 / UYR58262.1
Yezo virus (T-IM01)	S	ON563279.1 / UYR58263.1
Yezo virus (T-HLJ03)	L	ON563274.1 / UYR58258.1
Yezo virus (T-HLJ03)	M	ON563275.1 / UYR58259.1
Yezo virus (T-HLJ03)	S	ON563276.1 / UYR58260.1
Yezo virus (T-HLJ02)	L	ON563271.1 / UYR58255.1
Yezo virus (T-HLJ02)	M	ON563272.1 / UYR58256.1
Yezo virus (T-HLJ02)	S	ON563273.1 / UYR58257.1
Yezo virus (T-HLJ01)	L	ON563268.1 / UYR58252.1
Yezo virus (T-HLJ01)	M	ON563269.1 / UYR58253.1
Yezo virus (T-HLJ01)	S	ON563270.1 / UYR58254.1
Yezo virus (HH003-2020)	L	NC 079099.1 / YP 010840880.1
Yezo virus (HH003-2020)	M	NC 079098.1 / YP 010840879.1
Yezo virus (HH003-2020)	S	NC 079100.1 / YP 010840881.1
Yezo virus (BT-2155)	L	LC790680.1 / BEV30402.1
Yezo virus (BT-2155)	M	LC790681.1 / BEV30403.1
Yezo virus (BT-2155)	S	LC790682.1 / BEV30404.1
Yezo virus (BT-2135)	L	LC790677.1 / BEV30399.1
Yezo virus (BT-2135)	M	LC790678.1 / BEV30400.1
Yezo virus (BT-2135)	S	LC790679.1 / BEV30401.1
Yezo virus (BT-1968)	L	LC790674.1 / BEV30396.1
Yezo virus (BT-1968)	M	LC790675.1 / BEV30397.1
Yezo virus (BT-1968)	S	LC790676.1 / BEV30398.1
Yezo virus (BT-1864)	L	LC735734.1 / BDT53108.1
Yezo virus (BT-1864)	M	LC735735.1 / BDT53109.1
Yezo virus (BT-1864)	S	LC735736.1 / BDT63110.1
Yezo virus (BT-1844)	L	LC735731.1 / BDT53105.1

Yezo virus (BT-1844)	M	LC735732.1 / BDT53106.1
Yezo virus (BT-1844)	S	LC735733.1 / BDT53107.1
Yezo virus (BT-1826)	L	LC735728.1 / BDT53102.1
Yezo virus (BT-1826)	M	LC735729.1 / BDT53103.1
Yezo virus (BT-1826)	S	LC735730.1 / BDT53104.1
Yezo virus (BT-1821)	L	LC735725.1 / BDT53099.1
Yezo virus (BT-1821)	M	LC735726.1 / BDT53100.1
Yezo virus (BT-1821)	S	LC735727.1 / BDT53101.1
Sulina virus (b31)	L	PP260006.1
Sulina virus (b31)	M	PP260007.1
Sulina virus (b31)	S	PP260008.1
Sulina virus (Alsace)	L	OR613124.1
Sulina virus (Alsace)	M	OR613123.1
Sulina virus (Alsace)	S	OR613122.1
Sulina virus (IxriSL 16-01)	L	NC 078999.1
Sulina virus (IxriSL 16-01)	M	NC 078997.1
Sulina virus (IxriSL 16-01)	S	NC 078998.1

Table S2. Viruses and GenBank accession numbers of amino acid sequences of RdRp of species of the genus *Orhonairovirus* used in phylogenetic analyses.

Virus (strain or isolate)	Position of the virus within the genus *Otrhonairovirus*	GenBank accession number
Abu Hammad virus	member species	AMT75371.1
Abu Mina virus	member species	AMT75374.1
Aigai virus	member species	ABB30012.1
Antu virus	related, unclassified virus	WGL08458.1
Artashat virus	member species	AKC89358.1
Avalon virus	member species	AMT75377.1
Bandia virus (IPD/A611)	member species	NC 078253.1
Bandia virus (RV611)	member species	AMT75383.1
Burana virus	member species	AKC89349.1
Caspiy virus	member species	AKC89346.1
cencurut virus	related, unclassified virus	WAX23731.1
Chim virus	member species	AKC89343.1
Clo Mor virus	member species	KU343163.1
Clo Mor virus (ScotAr7)	member species	KU343139.1
Crimean-Congo hemorrhagic fever virus	member species	AAR25663.1
Dera Ghazi Khan virus	member species	KU343151.1
Dugbe virus	member species	AAB18834.1
Erve virus	member species	AFH89032.1
Estero Real virus	member species	AXP33563.1
Farralon virus	member species	KU343154.1
Finch creek virus	related, unclassified virus	ULT85602.1
Geran virus	member species	AKC89340.1
Gossas virus	member species	ALD83626.1
Great Saltee virus	member species	AMT75404.1
Gubbo nairovirus	related, unclassified virus	UZO29916.1
Hazara virus	member species	AJW66841.1
Huangpi tick virus 1	member species	AJG39237.1
Hughes virus	member species	AMT75407.1

Issyk-Kul virus	member species	AII79373.1
Kasokero virus	member species	ALD84349.1
Keterah virus	member species	ALD84352.1
Kupe virus	member species	ABY82502.1
Lamusara virus	related, unclassified virus	BDE11047.1
Leopards Hill virus	member species	BAP90965.1
Meihua Mountain virus	related, unclassified virus	UQM93876.1
Meram virus	member species	QNH88011.1
Nairobi sheep disease virus	member species	AIZ00432.1
Orthonairovirus sp. isolate YS	related, unclassified virus	UCR91749.1
Pacific coast tick nairovirus	member species	ARF07704.1
Pangolin orthonairovirus	related, unclassified virus	URZ29345.1
Paramushir virus	related, unclassified virus	AZB49553.1
Punta Salinas virus	member species	AMT75410.1
Qalyub virus	member species	KU343160.1
Raza virus	member species	AMT75416.1
Sakhalin virus	member species	AMT75419.1
Sapphire II virus	member species	QLA46845.1
Sapphire II virus (RML 52323-14)	member species	AMT75422.1
Soldado virus	member species	AMT75425.1
Songling virus	member species	QPO14991.1
Sulina virus	member species	QRR19149.1
Tacheng tick virus 1	member species	AJG39253.1
Taggert virus	member species	AMR73395.1
Tamdy virus (TT1)	member species	QNH88007.1
Tamdy virus (XJ01)	member species	QFU19352.1
Thiafora virus	member species	ALD84355.1
Tillamook virus	member species	AMT75431.1
Tofla virus	member species	BAU21075.1
Tunis virus	member species	AMT75434.1
Uzun-Agach virus	member species	AKC89313.1
Vinegar Hill virus	member species	AUD40046.1
Wenzhou tick virus	member species	AJG39255.1
Wufeng Crocidura attenuata orthonairovirus 1	related, unclassified virus	UOL48895.1
Yogue virus	member species	ALD84358.1
Zirqa virus (A2070-1)	member species	KU343169.1
Zirqa virus (Por 7866)	member species	AMT75437.1

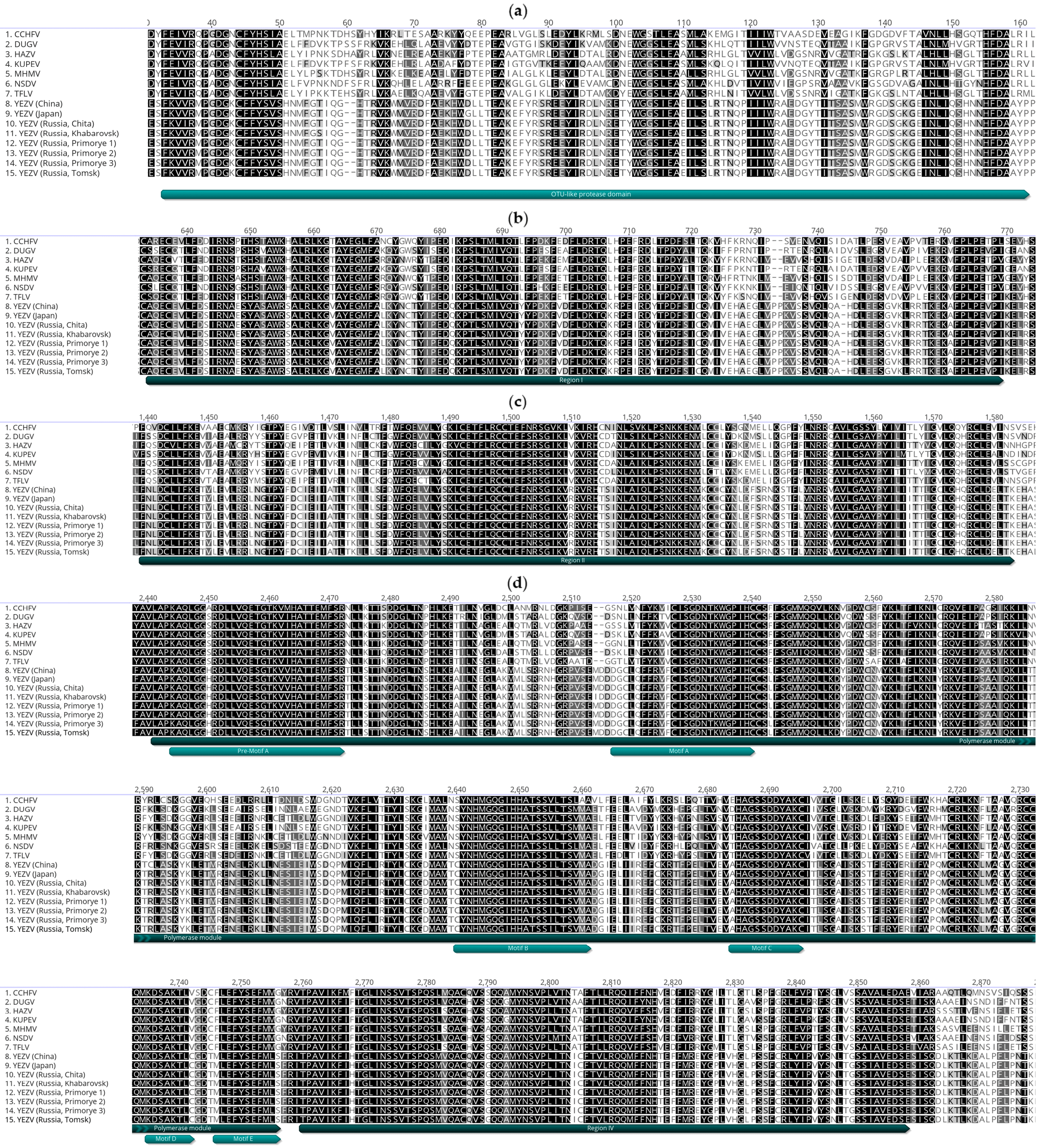

Figure S1. Amino acid sequence alignment of the conserved regions of NSD viruses and YEZV isolates. (**a**) OUT-like protease domain; (**b**) Region I; (**c**) Region II; (**d**) Polymerase module (pre-Motif A and Motif A-E) and region IV are highlighted and highly conserved in bunyaviruses. In order for a column to be rendered black (100% similar) all pairs of sites in the column must have a score (according to the specified score matrix Blosum62 and threshold = 1) equal to or exceeding the specified threshold. Thus, black indicates 100% similarity, dark grey - from 80% to 100% similarity, grey - from 60% to 80% similarity and light grey - less than 60% similarity. The selected orthonairoviruses: CCHFV – Crimean-Congo hemorrhagic fever virus (*O. haemorrhagiae*), DUGV – Dugbe virus (*O. dugbeense*), HAZV – Hazara virus (*O. hazaraense*), KUPEV – Kupe virus (*O. amblyommae*), MHMV – Meihua Mountain virus, NSDV – Nairobi sheep disease virus (*O. nairobiense*), TFLV – Tofla virus (*O. japonicum*).

Table S3. Amino acid substitutions in the RNA-dependent RNA polymerase (L segment) of the new YEZV genetic variant compared to Japanese and Russian YEZV isolates.

Sequence	Position / amino acid																		
	3	164	170	177	223	226	229	369	495	965	973	990	994	1014	1418	1441	1453	1486	1660
Tomsk_Ipav1	Ser	Ser	Thr	Val	Val	Ile	Ala	Ile	Asp	Ile	Val	Ile	Lys	Thr	Leu	Val	Met	Val	Val
XPK55871.1|Khabarovsk 2024-1	Gly	Gly	Thr	Thr	Met	Val	Val	Val	Glu	Ile	Ile	Val	Arg	Ala	Ser	Ala	Leu	Ala	Ala
XPK55870.1|Chita 2024-1	Gly	Gly	Ala	Thr	Met	Val	Val	Val	Glu	Ile	Ile	Val	Arg	Ala	Ser	Ala	Met	Ala	Val
XPK55869.1|Primorye 2024-3	Gly	Gly	Ala	Thr	Met	Val	Val	Val	Glu	Val	Ile	Val	Arg	Ala	Ser	Ala	Leu	Ala	Ala
XPK55868.1|Primorye 2024-2	Gly	Gly	Ala	Thr	Met	Val	Val	Val	Glu	Ile	Ile	Val	Arg	Ala	Ser	Ala	Leu	Ala	Val
XPK55867.1|Primorye 2024-1	Gly	Gly	Ala	Thr	Met	Val	Val	Val	Glu	Ile	Ile	Val	Arg	Ala	Ser	Ala	Leu	Ala	Val
BDT53100.1|BT-1821	Gly	Gly	Ala	Thr	Met	Val	Val	Val	Glu	Val	Ile	Val	Arg	Ala	Ser	Ala	Leu	Ala	Ala
BDT53103.1|BT-1826	Gly	Gly	Ala	Thr	Met	Val	Val	Val	Glu	Val	Ile	Val	Arg	Ala	Ser	Ala	Leu	Ala	Ala
BDT53106.1|BT-1844	Gly	Gly	Ala	Thr	Met	Val	Val	Val	Glu	Val	Ile	Val	Arg	Ala	Ser	Ala	Leu	Ala	Ala
BDT53109.1|BT-1864	Gly	Gly	Ala	Thr	Met	Val	Val	Val	Glu	Val	Ile	Val	Arg	Ala	Ser	Ala	Leu	Ala	Ala
BEV30397.1|BT-1968	Gly	Gly	Ala	Thr	Met	Val	Val	Val	Glu	Val	Ile	Val	Arg	Ala	Ser	Ala	Leu	Ala	Ala
BEV30400.1|BT-2135	Gly	Gly	Ala	Thr	Met	Val	Val	Val	Glu	Val	Ile	Val	Arg	Ala	Ser	Ala	Leu	Ala	Ala
BEV30403.1|BT-2155	Gly	Gly	Ala	Thr	Met	Val	Val	Val	Glu	Val	Ile	Val	Arg	Ala	Ser	Ala	Leu	Ala	Ala
YP 010840879.1|HH003-2020	Gly	Gly	Ala	Thr	Met	Val	Val	Val	Glu	Val	Ile	Val	Arg	Ala	Ser	Ala	Leu	Ala	Ala

Sequence	Position / amino acid																		
	1887	1985	1995	1998	2005	2088	2095	2110	2117	2123	2788	2797	2805	2807	2819	2826	3007	3160	3476
Tomsk_Ipav1	Gly	Arg	Asn	Thr	Lys	Lys	Arg	Lys	Leu	Ser	Leu	Val	Arg	Met	Ile	Val	Thr	Pro	His
XPK55871.1|Khabarovsk 2024-1	Ser	Lys	Ser	Ala	Thr	Arg	Met	Lys	Ser	Pro	Phe	Ile	Lys	Leu	Val	Ile	Ser	Ser	Tyr
XPK55870.1|Chita 2024-1	Ser	Lys	Ser	Ala	Thr	Arg	Met	Lys	Ser	Pro	Phe	Ile	Lys	Leu	Val	Ile	Ser	Ser	Tyr
XPK55869.1|Primorye 2024-3	Ser	Lys	Ser	Ala	Thr	Lys	Met	Arg	Ser	Pro	Phe	Ile	Lys	Leu	Val	Ile	Ser	Ser	Tyr
XPK55868.1|Primorye 2024-2	Ser	Lys	Ser	Ala	Thr	Arg	Met	Lys	Ser	Pro	Phe	Ile	Lys	Leu	Val	Ile	Ser	Ser	His
XPK55867.1|Primorye 2024-1	Ser	Lys	Ser	Ala	Thr	Arg	Met	Lys	Ser	Pro	Phe	Ile	Lys	Leu	Val	Ile	Ser	Ser	His
BDT53100.1|BT-1821	Ser	Lys	Ser	Ala	Thr	Arg	Met	Arg	Ser	Pro	Phe	Ile	Lys	Leu	Val	Ile	Ser	Ser	Tyr
BDT53103.1|BT-1826	Ser	Lys	Ser	Ala	Thr	Arg	Met	Arg	Ser	Pro	Phe	Ile	Lys	Leu	Val	Ile	Ser	Ser	Tyr
BDT53106.1|BT-1844	Ser	Lys	Ser	Ala	Thr	Arg	Met	Arg	Ser	Pro	Phe	Ile	Lys	Leu	Val	Ile	Ser	Ser	Tyr
BDT53109.1|BT-1864	Ser	Lys	Ser	Ala	Thr	Arg	Met	Arg	Ser	Pro	Phe	Ile	Lys	Leu	Val	Ile	Ser	Ser	Tyr
BEV30397.1|BT-1968	Ser	Lys	Ser	Ala	Thr	Arg	Met	Arg	Ser	Pro	Phe	Ile	Lys	Leu	Val	Ile	Ser	Ser	Tyr
BEV30400.1|BT-2135	Ser	Lys	Ser	Ala	Thr	Arg	Met	Arg	Ser	Pro	Phe	Ile	Lys	Leu	Val	Ile	Ser	Ser	Tyr
BEV30403.1|BT-2155	Ser	Lys	Ser	Ala	Thr	Arg	Met	Arg	Ser	Pro	Phe	Ile	Lys	Leu	Val	Ile	Ser	Ser	Tyr
YP 010840879.1|HH003-2020	Ser	Lys	Ser	Ala	Thr	Arg	Met	Arg	Ser	Pro	Phe	Ile	Lys	Leu	Val	Ile	Ser	Ser	Tyr

Sequence	Position / amino acid						
	3566	3743	3758	3762	3826	3851	3926
Tomsk_Ipav1	Ala	Tyr	Asn	Cys	Ser	Ser	Arg
XPK55871.1|Khabarovsk 2024-1	Ser	His	Ser	Asp	Asn	Gly	Lys
XPK55870.1|Chita 2024-1	Ser	His	Asn	Asp	Asn	Gly	Lys
XPK55869.1|Primorye 2024-3	Ser	His	Ser	Asp	Asn	Gly	Lys
XPK55868.1|Primorye 2024-2	Ser	His	Ser	Asp	Asn	Gly	Lys
XPK55867.1|Primorye 2024-1	Ser	His	Ser	Asp	Asn	Gly	Lys
BDT53100.1|BT-1821	Ser	His	Ser	Asp	Asn	Gly	Arg
BDT53103.1|BT-1826	Ser	His	Ser	Asp	Asn	Gly	Arg
BDT53106.1|BT-1844	Ser	His	Ser	Asp	Asn	Gly	Arg
BDT53109.1|BT-1864	Ser	His	Ser	Asp	Asn	Gly	Lys
BEV30397.1|BT-1968	Ser	His	Ser	Asp	Asn	Gly	Lys
BEV30400.1|BT-2135	Ser	His	Ser	Asp	Asn	Gly	Lys
BEV30403.1|BT-2155	Ser	His	Ser	Asp	Asn	Gly	Arg
YP 010840879.1|HH003-2020	Ser	His	Ser	Asp	Asn	Gly	Lys

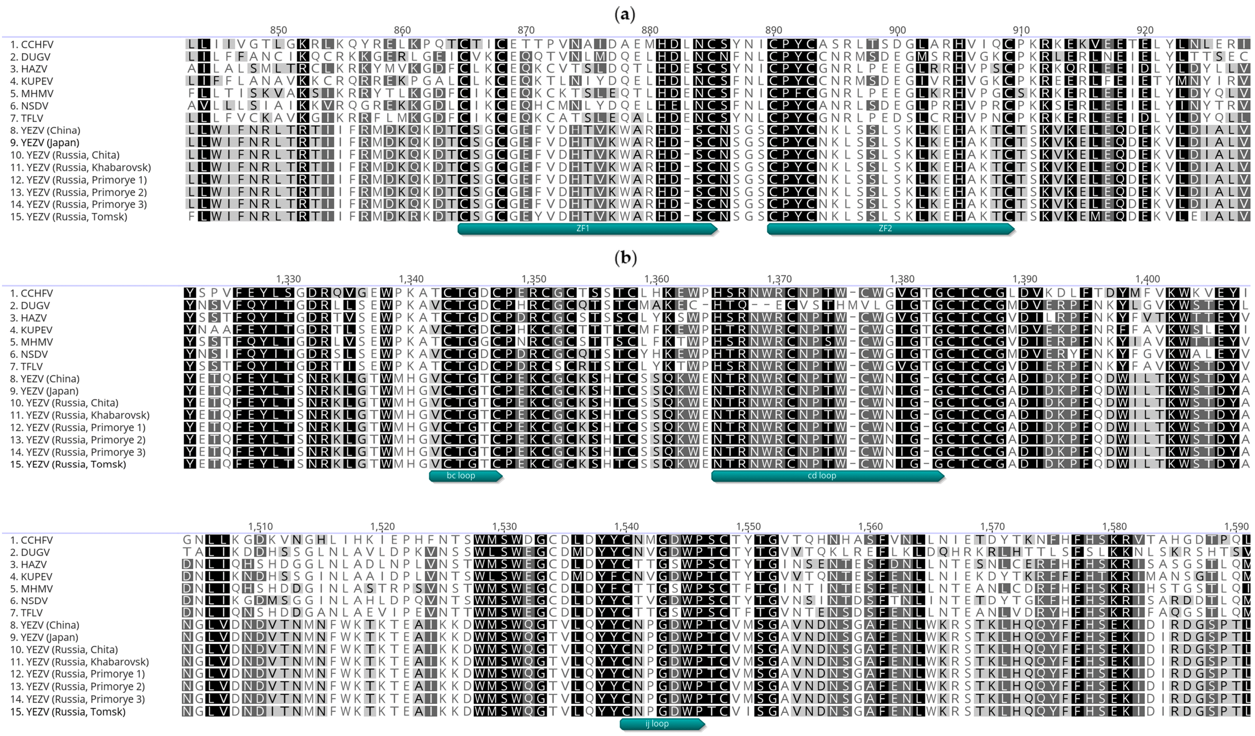

Figure S2. Amino acid sequence alignment of the Gns and Gcs of NSD genogroup viruses and YEZV isolates. (**a**) Glycoprotein Gn, two C-terminus localized zinc-finger motifs are highlighted; (**b**) Glycoprotein Gc, fusion loops are highlighted. In order for a column to be rendered black (100% similar) all pairs of sites in the column must have a score (according to the specified score matrix Blosum62 and threshold = 1) equal to or exceeding the specified threshold. Thus, black indicates 100% similarity, dark grey - from 80% to 100% similarity, grey - from 60% to 80% similarity and light grey - less than 60% similarity. The selected orthonairoviruses: CCHFV – Crimean-Congo hemorrhagic fever virus (*O. haemorrhagiae*), DUGV – Dugbe virus (*O. dugbeense*), HAZV – Hazara virus (*O. hazaraense*), KUPEV – Kupe virus (*O. amblyommae*), MHMV – Meihua Mountain virus, NSDV – Nairobi sheep disease virus (*O. nairobiense*), TFLV – Tofla virus (*O. japonicum*).

Table S4. Amino acid substitutions in the glycoprotein complex (M segment) of the new YEZV genetic variant compared to Japanese and Russian YEZV isolates.

**Sequence**	**Position / amino acid**																				
	3	6	8	9	11	14	20	28	29	30	32	41	45	47	53	58	60	62	83	100	103
Tomsk_Ipav1	Asn	Tyr	Gly	Ile	Thr	Ala	Arg	Pro	Gly	Ala	Ile	Ala	Gly	Ala	Ser	Pro	Ala	Thr	Ile	Val	Ala
XPK55871.1|Khabarovsk 2024-1	Ile	Cys	Asp	Phe	Ala	Leu	Leu	Leu	Asn	Thr	Val	Thr	Ser	Val	Thr	Thr	Thr	Ala	Leu	Thr	Val
XPK55870.1|Chita 2024-1	Ile	Tyr	Asp	Phe	Ala	Leu	Leu	Leu	Asn	Thr	Val	Thr	Ser	Val	Thr	Thr	Thr	Thr	Leu	Thr	Val
XPK55869.1|Primorye 2024-3	Ile	Cys	Asp	Phe	Ala	Leu	Leu	Leu	Asn	Thr	Val	Thr	Ser	Val	Thr	Thr	Thr	Ala	Leu	Thr	Val
XPK55868.1|Primorye 2024-2	Ile	Cys	Asn	Phe	Ala	Leu	Leu	Leu	Asn	Thr	Val	Thr	Ser	Val	Thr	Thr	Thr	Ala	Leu	Thr	Val
XPK55867.1|Primorye 2024-1	Ile	Cys	Asn	Phe	Ala	Leu	Leu	Leu	Asn	Thr	Val	Thr	Ser	Val	Thr	Thr	Thr	Ala	Leu	Thr	Val
BDT53100.1|BT-1821	Ile	Cys	Asp	Phe	Ala	Leu	Leu	Leu	Asn	Thr	Val	Thr	Ser	Val	Thr	Thr	Thr	Ala	Leu	Thr	Val
BDT53103.1|BT-1826	Ile	Cys	Asp	Phe	Ala	Leu	Leu	Leu	Asn	Thr	Val	Thr	Ser	Val	Thr	Thr	Thr	Ala	Leu	Thr	Val
BDT53106.1|BT-1844	Ile	Cys	Asp	Phe	Ala	Leu	Leu	Leu	Asn	Thr	Val	Thr	Ser	Val	Thr	Thr	Thr	Ala	Leu	Thr	Val
BDT53109.1|BT-1864	Ile	Cys	Asp	Phe	Ala	Leu	Leu	Leu	Asn	Thr	Val	Thr	Ser	Val	Thr	Thr	Thr	Ala	Leu	Thr	Val
BEV30397.1|BT-1968	Ile	Cys	Asp	Phe	Ala	Leu	Leu	Leu	Asn	Thr	Ile	Thr	Ser	Val	Thr	Thr	Thr	Ala	Leu	Thr	Val
BEV30400.1|BT-2135	Ile	Cys	Asp	Phe	Ala	Leu	Leu	Leu	Asn	Thr	Val	Thr	Ser	Val	Thr	Thr	Thr	Thr	Leu	Thr	Val
BEV30403.1|BT-2155	Ile	Cys	Asp	Phe	Ala	Leu	Leu	Leu	Asn	Thr	Val	Thr	Ser	Val	Thr	Thr	Thr	Ala	Leu	Thr	Val
YP 010840879.1|HH003-2020	Ile	Cys	Asp	Phe	Ala	Leu	Leu	Leu	Asn	Thr	Val	Thr	Ser	Val	Thr	Thr	Thr	Ala	Leu	Thr	Val

Sequence	Position / amino acid																			
	115	121	124	157	174	186	213	258	263	314	349	479	491	520	522	537	547	552	553	554
Tomsk_Ipav1	Thr	Val	Thr	Lys	Ile	Ala	Leu	Ser	Asn	Val	Pro	Asn	Arg	Asn	Phe	Lys	Ile	Leu	Val	Gly
XPK55871.1|Khabarovsk 2024-1	Asn	Gly	Ser	Arg	Val	Thr	Arg	Gly	Asp	Ala	His	Ile	Lys	Ser	Leu	Arg	Val	Ile	Gly	Arg
XPK55870.1|Chita 2024-1	Asn	Gly	Ser	Arg	Val	Thr	Arg	Gly	Asp	Ala	His	Ile	Lys	Ser	Leu	Arg	Val	Ile	Gly	Arg
XPK55869.1|Primorye 2024-3	Asn	Gly	Ser	Arg	Val	Thr	Arg	Gly	Asp	Ala	His	Ile	Lys	Ser	Leu	Arg	Val	Ile	Gly	Arg
XPK55868.1|Primorye 2024-2	Asn	Gly	Ser	Arg	Val	Thr	Arg	Gly	Asp	Ala	His	Ile	Lys	Ser	Leu	Arg	Val	Ile	Gly	Arg
XPK55867.1|Primorye 2024-1	Asn	Gly	Ser	Arg	Val	Thr	Arg	Gly	Asp	Ala	His	Ile	Lys	Ser	Leu	Arg	Val	Ile	Gly	Arg
BDT53100.1|BT-1821	Asn	Gly	Ser	Arg	Val	Thr	Arg	Gly	Asp	Ala	His	Ile	Lys	Ser	Leu	Arg	Val	Ile	Gly	Arg
BDT53103.1|BT-1826	Asn	Gly	Ser	Arg	Val	Thr	Arg	Gly	Asp	Ala	His	Ile	Lys	Ser	Leu	Arg	Val	Ile	Gly	Arg
BDT53106.1|BT-1844	Asn	Gly	Ser	Arg	Val	Thr	Arg	Gly	Asp	Ala	His	Ile	Lys	Ser	Leu	Arg	Val	Ile	Gly	Arg
BDT53109.1|BT-1864	Asn	Gly	Ser	Arg	Val	Thr	Arg	Gly	Asp	Ala	His	Ile	Lys	Ser	Leu	Arg	Val	Ile	Gly	Arg
BEV30397.1|BT-1968	Asn	Gly	Ser	Arg	Val	Thr	Arg	Gly	Asp	Ala	His	Ile	Lys	Ser	Leu	Arg	Val	Ile	Gly	Arg
BEV30400.1|BT-2135	Asn	Gly	Ser	Arg	Val	Thr	Arg	Gly	Asp	Ala	His	Ile	Lys	Ser	Leu	Arg	Val	Ile	Gly	Arg
BEV30403.1|BT-2155	Asn	Gly	Ser	Arg	Val	Thr	Arg	Gly	Asp	Ala	His	Ile	Lys	Ser	Leu	Arg	Val	Ile	Gly	Arg
YP 010840879.1|HH003-2020	Asn	Gly	Ser	Arg	Val	Thr	Arg	Gly	Asp	Ala	His	Ile	Lys	Ser	Leu	Arg	Val	Ile	Gly	Arg

Sequence	Position / amino acid																			
	555	556	557	558	561	562	563	581	591	635	643	656	672	711	728	729	730	810	978	989
Tomsk_Ipav1	Leu	His	Leu	Phe	Ser	Phe	Phe	Arg	Tyr	Met	Glu	Tyr	Ile	Ile	Asp	Ala	Gln	Met	Ser	Asp
XPK55871.1|Khabarovsk 2024-1	Ile	Ala	Phe	Ile	Phe	Leu	Leu	Gln	Phe	Leu	Asp	Ser	Val	Val	Thr	Leu	His	Leu	Asn	Gly
XPK55870.1|Chita 2024-1	Ile	Ala	Phe	Ile	Phe	Leu	Leu	Gln	Phe	Leu	Asp	Ser	Val	Val	Thr	Val	His	Leu	Asn	Gly
XPK55869.1|Primorye 2024-3	Ile	Ala	Phe	Ile	Phe	Leu	Leu	Gln	Phe	Leu	Asp	Ser	Val	Val	Ala	Leu	His	Leu	Asn	Gly
XPK55868.1|Primorye 2024-2	Ile	Ala	Phe	Ile	Phe	Leu	Leu	Gln	Phe	Leu	Asp	Ser	Val	Val	Thr	Val	His	Leu	Asn	Gly
XPK55867.1|Primorye 2024-1	Ile	Ala	Phe	Ile	Phe	Leu	Leu	Gln	Phe	Leu	Asp	Ser	Val	Val	Thr	Val	His	Leu	Asn	Gly
BDT53100.1|BT-1821	Ile	Ala	Phe	Ile	Phe	Leu	Leu	Gln	Phe	Leu	Asp	Ser	Val	Val	Thr	Leu	His	Leu	Asn	Gly
BDT53103.1|BT-1826	Ile	Ala	Phe	Ile	Phe	Leu	Leu	Gln	Phe	Leu	Asp	Ser	Val	Val	Thr	Leu	His	Leu	Asn	Gly
BDT53106.1|BT-1844	Ile	Ala	Phe	Ile	Phe	Leu	Leu	Gln	Phe	Leu	Asp	Ser	Val	Val	Thr	Leu	His	Leu	Asn	Gly
BDT53109.1|BT-1864	Ile	Ala	Phe	Ile	Phe	Leu	Leu	Gln	Phe	Leu	Asp	Ser	Val	Val	Thr	Leu	His	Leu	Asn	Gly
BEV30397.1|BT-1968	Ile	Ala	Phe	Ile	Phe	Leu	Leu	Gln	Phe	Leu	Asp	Ser	Val	Val	Thr	Leu	His	Leu	Asn	Gly
BEV30400.1|BT-2135	Ile	Ala	Phe	Ile	Phe	Leu	Leu	Gln	Phe	Leu	Asp	Ser	Val	Val	Thr	Val	His	Leu	Asn	Gly
BEV30403.1|BT-2155	Ile	Ala	Phe	Ile	Phe	Leu	Leu	Gln	Phe	Leu	Asp	Ser	Val	Val	Thr	Leu	His	Leu	Asn	Gly
YP 010840879.1|HH003-2020	Ile	Ala	Phe	Ile	Phe	Leu	Leu	Gln	Phe	Leu	Asp	Ser	Val	Val	Thr	Leu	His	Leu	Asn	Gly

Sequence	Position / amino acid																		
	991	1023	1062	1224	1255	1279	1289	1291	1310	1313	1320	1322	1325	1332	1333	1339	1342	1347	1350
Tomsk_Ipav1	Val	Ile	Ile	Leu	Arg	Tyr	Ile	Val	Ile	Ile	Asn	Met	Arg	Arg	Lys	Lys	Val	Ala	Met
XPK55871.1|Khabarovsk 2024-1	Ile	Val	Met	Ser	Lys	Phe	Val	Ile	Val	Val	Ser	Ile	Lys	Lys	Arg	Arg	Ile	Glu	Arg
XPK55870.1|Chita 2024-1	Ile	Val	Met	Ser	Lys	Phe	Val	Ile	Val	Val	Ser	Ile	Lys	Lys	Arg	Arg	Ile	Glu	Arg
XPK55869.1|Primorye 2024-3	Ile	Val	Met	Ser	Lys	Phe	Val	Ile	Val	Val	Ser	Ile	Lys	Lys	Arg	Arg	Ile	Glu	Arg
XPK55868.1|Primorye 2024-2	Ile	Val	Met	Ser	Lys	Phe	Val	Ile	Val	Val	Ser	Ile	Lys	Lys	Arg	Arg	Ile	Glu	Arg
XPK55867.1|Primorye 2024-1	Ile	Val	Met	Ser	Lys	Phe	Val	Ile	Val	Val	Ser	Ile	Lys	Lys	Arg	Arg	Ile	Glu	Arg
BDT53100.1|BT-1821	Ile	Val	Met	Ser	Lys	Phe	Val	Ile	Val	Val	Ser	Ile	Lys	Lys	Arg	Arg	Ile	Glu	Arg
BDT53103.1|BT-1826	Ile	Val	Met	Ser	Lys	Phe	Val	Ile	Val	Val	Ser	Ile	Lys	Lys	Arg	Arg	Ile	Glu	Arg
BDT53106.1|BT-1844	Ile	Val	Met	Ser	Lys	Phe	Val	Ile	Val	Val	Ser	Ile	Lys	Lys	Arg	Arg	Ile	Glu	Arg
BDT53109.1|BT-1864	Ile	Val	Met	Ser	Lys	Phe	Val	Ile	Val	Val	Ser	Ile	Lys	Lys	Arg	Arg	Ile	Glu	Arg
BEV30397.1|BT-1968	Ile	Val	Met	Ser	Lys	Phe	Val	Ile	Val	Val	Ser	Ile	Lys	Lys	Arg	Arg	Ile	Glu	Arg
BEV30400.1|BT-2135	Ile	Val	Met	Ser	Lys	Phe	Val	Ile	Val	Val	Ser	Ile	Lys	Lys	Arg	Arg	Ile	Glu	Arg
BEV30403.1|BT-2155	Ile	Val	Met	Ser	Lys	Phe	Val	Ile	Val	Val	Ser	Ile	Lys	Lys	Arg	Arg	Ile	Glu	Arg
YP 010840879.1|HH003-2020	Ile	Val	Met	Ser	Lys	Phe	Val	Ile	Val	Val	Ser	Ile	Lys	Lys	Arg	Arg	Ile	Glu	Arg

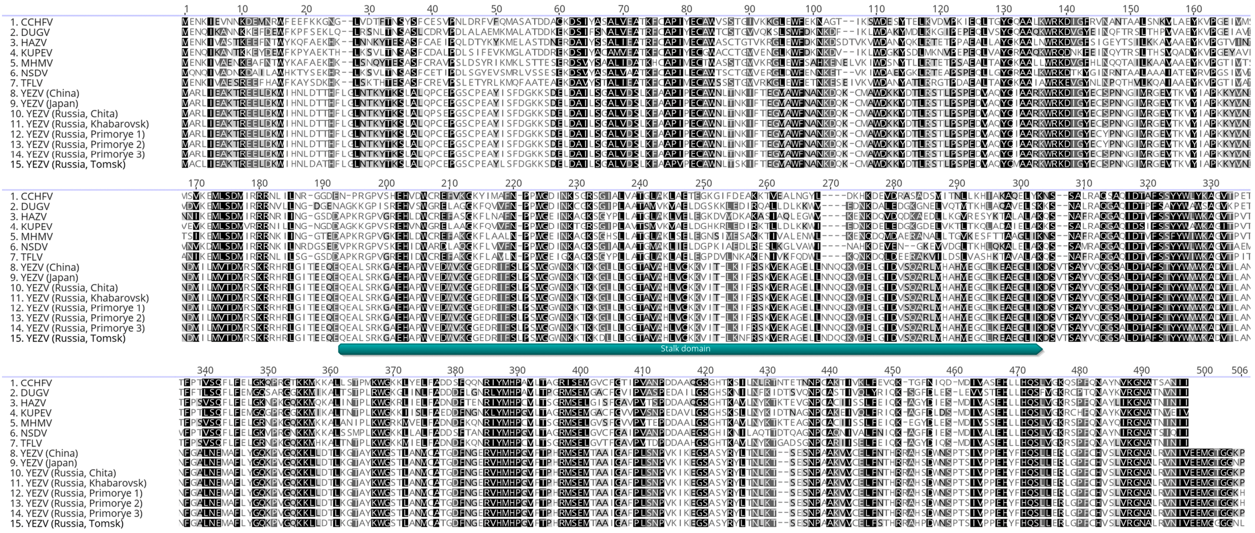

Figure S3. Amino acid sequence alignment of the nucleoproteins of NSD genogroup viruses and YEZV isolates. The flexible stalk domain is highlighted. In order for a column to be rendered black (100% similar) all pairs of sites in the column must have a score (according to the specified score matrix Blosum62 and threshold = 1) equal to or exceeding the specified threshold. Thus, black indicates 100% similarity, dark grey - from 80% to 100% similarity, grey - from 60% to 80% similarity and light grey - less than 60% similarity. The selected orthonairoviruses: CCHFV – Crimean-Congo hemorrhagic fever virus (*O. haemorrhagiae*), DUGV – Dugbe virus (*O. dugbeense*), HAZV – Hazara virus (*O. hazaraense*), KUPEV – Kupe virus (*O. amblyommae*), MHMV – Meihua Mountain virus, NSDV – Nairobi sheep disease virus (*O. nairobiense*), TFLV – Tofla virus (*O. japonicum*).

Table S5. Amino acid substitutions in the nucleoprotein (S segment) of the new YEZV genetic variant compared to Japanese and Russian YEZV isolates.

<table>
<tr><th rowspan="2">Sequence</th><th colspan="16">Position / amino acid</th></tr>
<tr><th>3</th><th>22</th><th>51</th><th>79</th><th>88</th><th>131</th><th>233</th><th>254</th><th>261</th><th>268</th><th>294</th><th>444</th><th>451</th><th>498</th><th>501</th><th>502</th></tr>
<tr><td>Tomsk_Ipav1</td><td>Cys</td><td>Ala</td><td>Leu</td><td>Val</td><td>Ser</td><td>Met</td><td>Asp</td><td>Asn</td><td>Arg</td><td>Asp</td><td>Arg</td><td>Ser</td><td>Pro</td><td>Gly</td><td>Asn</td><td>Leu</td></tr>
<tr><td>XPK55876.1|Khabarovsk 2024-1</td><td>Arg</td><td>Thr</td><td>Ile</td><td>Ile</td><td>Asn</td><td>Ile</td><td>Gly</td><td>Ile</td><td>Lys</td><td>Asn</td><td>Lys</td><td>Asn</td><td>Ser</td><td>Thr</td><td>Lys</td><td>Pro</td></tr>
<tr><td>XPK55875.1|Chita 2024-1</td><td>Arg</td><td>Thr</td><td>Ile</td><td>Ile</td><td>Asn</td><td>Ile</td><td>Gly</td><td>Ile</td><td>Arg</td><td>Asn</td><td>Lys</td><td>Asn</td><td>Ser</td><td>Thr</td><td>Lys</td><td>Pro</td></tr>
<tr><td>XPK55874.1|Primorye 2024-3</td><td>Arg</td><td>Thr</td><td>Ile</td><td>Ile</td><td>Asn</td><td>Ile</td><td>Gly</td><td>Ile</td><td>Lys</td><td>Asn</td><td>Lys</td><td>Asn</td><td>Ser</td><td>Thr</td><td>Lys</td><td>Pro</td></tr>
<tr><td>XPK55873.1|Primorye 2024-2</td><td>Arg</td><td>Thr</td><td>Ile</td><td>Ile</td><td>Asn</td><td>Ile</td><td>Gly</td><td>Ile</td><td>Lys</td><td>Asn</td><td>Lys</td><td>Asn</td><td>Ser</td><td>Thr</td><td>Lys</td><td>His</td></tr>
<tr><td>XPK55872.1|Primorye 2024-1</td><td>Arg</td><td>Thr</td><td>Ile</td><td>Ile</td><td>Asn</td><td>Ile</td><td>Gly</td><td>Ile</td><td>Lys</td><td>Asn</td><td>Lys</td><td>Asn</td><td>Ser</td><td>Thr</td><td>Lys</td><td>His</td></tr>
<tr><td>BDT53101.1|BT-1821</td><td>Arg</td><td>Thr</td><td>Ile</td><td>Ile</td><td>Asn</td><td>Ile</td><td>Gly</td><td>Ile</td><td>Lys</td><td>Asn</td><td>Lys</td><td>Asn</td><td>Ser</td><td>Thr</td><td>Asn</td><td>Pro</td></tr>
<tr><td>BDT53104.1|BT-1826</td><td>Arg</td><td>Thr</td><td>Ile</td><td>Ile</td><td>Asn</td><td>Ile</td><td>Gly</td><td>Ile</td><td>Lys</td><td>Asn</td><td>Lys</td><td>Asn</td><td>Ser</td><td>Thr</td><td>Asn</td><td>Pro</td></tr>
<tr><td>BDT53107.1|BT-1844</td><td>Arg</td><td>Thr</td><td>Ile</td><td>Ile</td><td>Asn</td><td>Ile</td><td>Gly</td><td>Ile</td><td>Lys</td><td>Asn</td><td>Lys</td><td>Asn</td><td>Ser</td><td>Thr</td><td>Asn</td><td>Pro</td></tr>
<tr><td>BDT53110.1|BT-1864</td><td>Arg</td><td>Thr</td><td>Ile</td><td>Ile</td><td>Asn</td><td>Ile</td><td>Gly</td><td>Ile</td><td>Lys</td><td>Asn</td><td>Lys</td><td>Asn</td><td>Ser</td><td>Thr</td><td>Asn</td><td>Pro</td></tr>
<tr><td>BEV30398.1|BT-1968</td><td>Arg</td><td>Thr</td><td>Ile</td><td>Ile</td><td>Asn</td><td>Ile</td><td>Gly</td><td>Ile</td><td>Lys</td><td>Asn</td><td>Lys</td><td>Asn</td><td>Ser</td><td>Thr</td><td>Asn</td><td>Pro</td></tr>
<tr><td>BEV30401.1|BT-2135</td><td>Arg</td><td>Thr</td><td>Ile</td><td>Ile</td><td>Asn</td><td>Ile</td><td>Gly</td><td>Ile</td><td>Lys</td><td>Asn</td><td>Lys</td><td>Asn</td><td>Ser</td><td>Thr</td><td>Asn</td><td>Pro</td></tr>
<tr><td>BEV30404.1|BT-2155</td><td>Arg</td><td>Thr</td><td>Ile</td><td>Ile</td><td>Asn</td><td>Ile</td><td>Gly</td><td>Ile</td><td>Lys</td><td>Asn</td><td>Lys</td><td>Asn</td><td>Ser</td><td>Thr</td><td>Asn</td><td>Pro</td></tr>
<tr><td>YP 010840881.1|HH003-2020</td><td>Arg</td><td>Thr</td><td>Ile</td><td>Ile</td><td>Asn</td><td>Ile</td><td>Gly</td><td>Ile</td><td>Lys</td><td>Asn</td><td>Lys</td><td>Asn</td><td>Ser</td><td>Thr</td><td>Asn</td><td>Pro</td></tr>
</table>

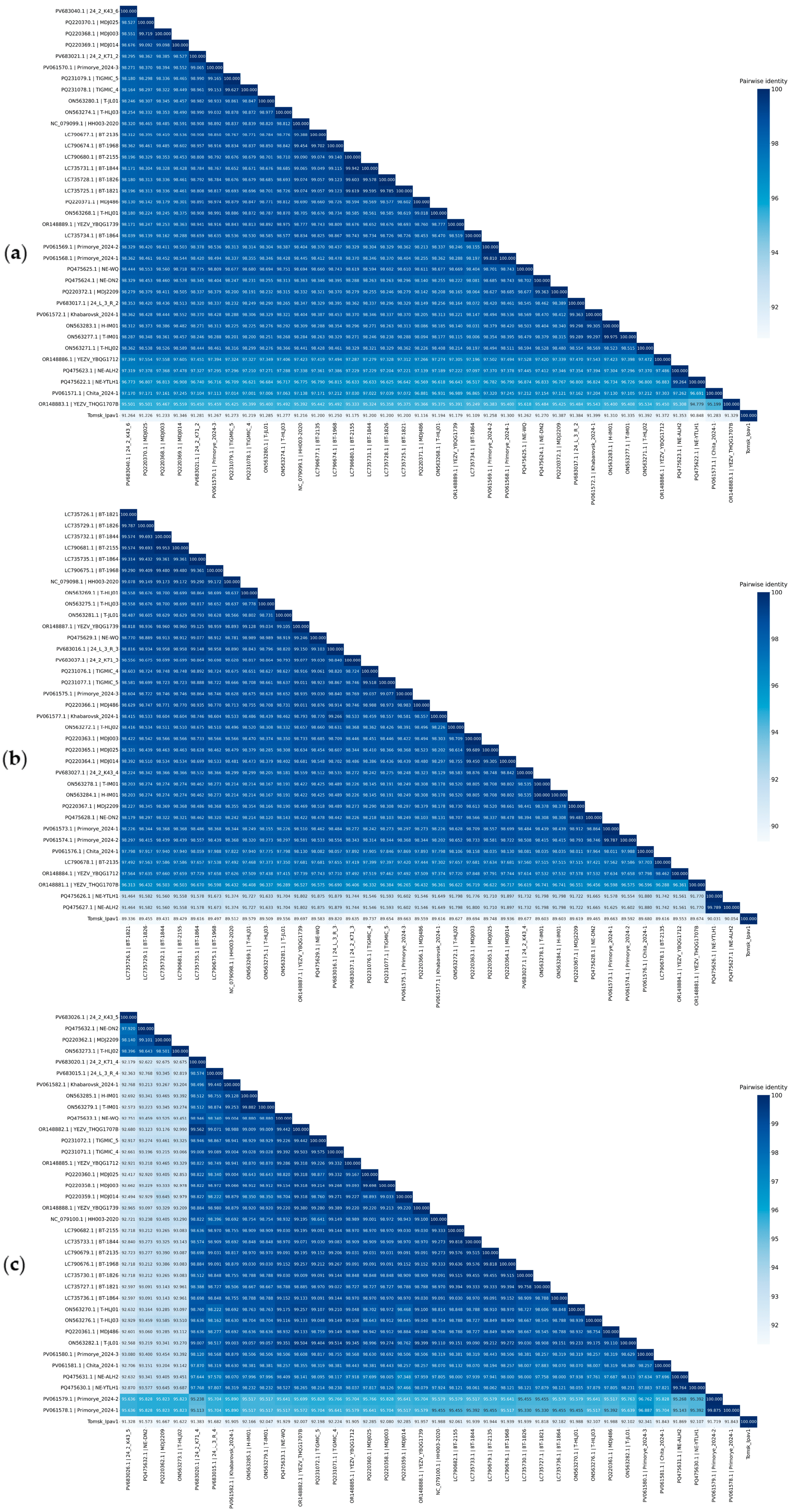

Figure S4. Pairwise nucleic acid identity matrix of YEZV isolates. (**a**) RdRp; (**b**) GPC; (**c**) Nucleoprotein. The gradient scale from blue to white shows the degree of sequence identity, with blue indicating a higher degree of identity and white indicating a lower degree of identity.

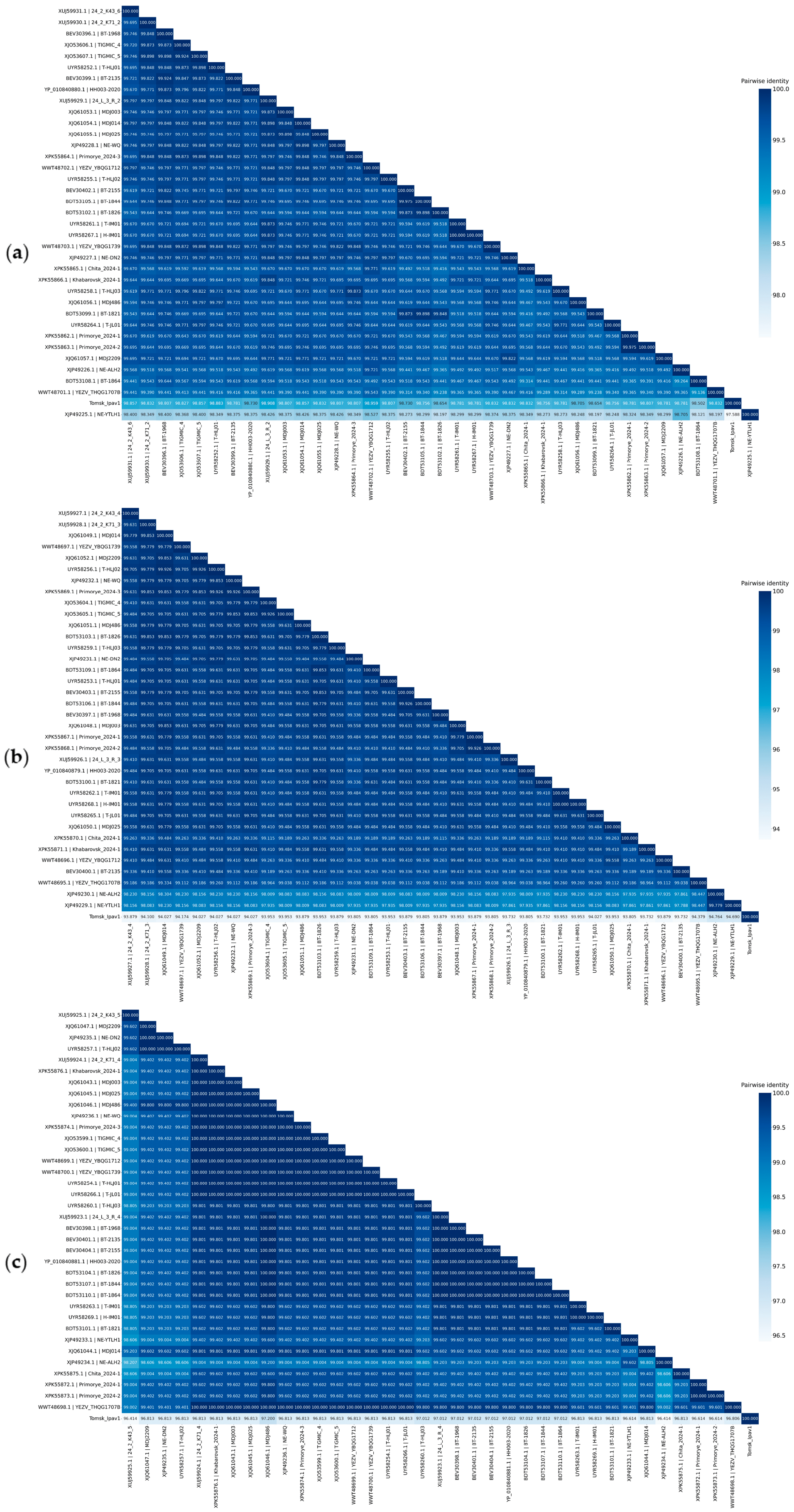

Figure S5. Pairwise amino acid identity matrix of YEZV isolates. (**a**) RdRp; (**b**) GPC; (**c**) Nucleoprotein. The gradient from blue to white shows the degree of sequence identity, with blue indicating a higher degree of identity and white indicating a lower degree of identity.
